# Identification of QTLs for yield and agronomic traits in rice under stagnant flooding conditions

**DOI:** 10.1186/s12284-017-0154-5

**Published:** 2017-04-20

**Authors:** Anshuman Singh, Jerome Carandang, Zennia Jean C. Gonzaga, Bertrand C. Y. Collard, Abdelbagi M. Ismail, Endang M. Septiningsih

**Affiliations:** 1 Present address: Rani Lakshmi Bai Central Agricultural University, Jhansi, 284003 India; 2 Present address: Sugar Research Australia, 71378 Bruce Highway, Meringa, PO Box122, Gordonvale, QLD 4865 Australia; 3 Present address: Department of Soil and Crop Sciences, Texas A&M University, College Station, TX 77843 USA; 4 International Rice Research Institute, DAPO 7777, Metro Manila, Philippines

**Keywords:** Irrigated rice, *Oryza sativa*, QTL, Rainfed rice, Stagnant flooding, Sub1 varieties

## Abstract

**Background:**

Stagnant flooding, where water of 25–50 cm remains until harvest time, is a major problem in rainfed lowland areas. Most of the Sub1 varieties, which can withstand around 2 weeks of complete submergence, perform poorly in these conditions. Hence, varieties tolerant of stagnant flooding are essential.

**Results:**

This paper presents the first study to map QTLs associated with tolerance to stagnant flooding, along with a parallel study under normal irrigation, using an F_7_ mapping population consisting of 148 RILs derived from a cross of Ciherang-Sub1 and the stagnant-flooding tolerant line IR10F365. Phenotypic data was collected for 15 key traits under both environments. Additionally, survival rate was measured under stress conditions. Genotyping was performed using the Illumina Infinium genotyping platform with a 6 K SNP chip, resulting in 469 polymorphic SNPs. Under stress and irrigated conditions, 38 and 46 QTLs were identified, respectively. Clusters of QTLs were detected in both stress and normal conditions, especially on chromosomes 3 and 5.

**Conclusions:**

Unique and common QTLs were identified and their physiological consequences are discussed. These beneficial QTLs can be used as targets for molecular breeding and can be further investigated to understand the underlying molecular mechanisms involved in stagnant flooding tolerance in rice.

**Electronic supplementary material:**

The online version of this article (doi:10.1186/s12284-017-0154-5) contains supplementary material, which is available to authorized users.

## Background

Most of rainfed lowlands and some irrigated areas in South and Southeast Asia are prone to flooding which causes huge crop losses worldwide. This situation may worsen under future climate change scenarios (Redfern et al. 2012). Floods are largely caused by incessant rainfall during the monsoon season from mid-June to early October, often causing complete submergence followed by stagnant flooding in areas with poor drainage, resulting in huge financial losses (Singh et al. 2011). Rice has developed adaptive mechanisms to survive under a wide range of water regimes; however, extreme weather leading to too much or too little water may cause serious yield reductions or total crop loss.

Different types of flooding can reduce rice production. Flash flooding is the most common, which occurs when rice plants are completely submerged for up to 2 weeks during the vegetative stage. If submergence prevails for more than 5 days, susceptible rice plants start to die and recovery is hampered (Mackill et al. 2010). Fortunately, a number of Sub1 varieties have been developed and released as remedies for this type of flooding, as the *Sub1* gene enables the submerged plants to become dormant and conserve resources for a rapid recovery after the floods recede. The first generation of Sub1 lines were the first six varieties developed by the International Rice Research Institute (IRRI), including Swarna-Sub1, IR64-Sub1, Samba Mahsuri-Sub1, BR11-Sub1, TDK1-Sub1, and CR1009-Sub1. While the second generation of Sub1 lines developed by IRRI were Ciherang-Sub1 and PSB Rc18-Sub1 in which IR64-Sub1 was used as the donor for the *SUB1* gene (Iftekharuddaula et al. 2011; 2016; Ismail et al. 2013; Neeraja et al. 2007; Septiningsih et al. 2009, 2015). Another type of flooding stress is flooding during germination, also known as anaerobic germination (AG). This can be due to unlevelled fields in direct-seeded rainfed or irrigated environments, especially in flood affected areas, when rain falls just after seeding causing poor seedling establishment (Ismail et al. 2009). Several major QTLs for tolerance to AG have been identified (Angaji et al. 2010; Baltazar et al. 2014; Septiningsih et al. 2013b) and a few lines with tolerance to AG alone or combined with *SUB1* have been developed through molecular and conventional breeding (Kretzschmar et al. 2015; Septiningsih et al. 2013a; Toledo et al. 2015). Some rainfed areas are also affected by deep water flooding where water stagnation is more than 1 m. In this situation, deepwater adapted varieties with fast internode elongation are required to escape from water while their shoot tips and tillers remain above the water (Catling 1992; Hattori et al. 2009). QTLs and genes have been identified and some tolerant lines have been developed for this type of flooding (Hattori et al. 2008, 2009).

The last type of flooding stress, which is the focus of this study, is stagnant flooding (SF), where water of 25 to 50 cm depth stagnates in the field for several weeks to few months (Mackill et al. 2010; Singh et al. 2011). In this stress environment, varieties with facultative elongation are needed; however, deepwater rice performs poorly under stagnant flooding conditions mainly due to lodging and the consequent reductions in yield and grain quality (Kato et al. 2014; Vergara et al. 2014). At the same time, most irrigated rice varieties also perform poorly under these conditions, as water depth greater than 25 cm greatly inhibits the growth of rice. The detrimental effect of SF can be seen as reduced number of tillers, poor fertility, smaller panicles and excessive lodging; together leading to poor grain yield and quality (Kato et al. 2014; Singh et al. 2014). The situation worsens when floods result in complete submergence followed by stagnant flooding until harvest time (Singh et al. 2011). Therefore, it is essential to develop varieties having tolerance of complete submergence during vegetative stage and stagnant flooding in the same genetic background. Farmers mostly use traditional low-yielding landraces in areas that are prone to both stresses within the same season. These landraces have partial tolerance of complete submergence and can resist stagnant flooding stress by stem and leaf elongation, but they have low yield of around 0.5–1.5 t ha^−1^ and low quality (Singh et al. 2011). The introgression of *SUB1* QTL in several mega varieties successfully improved submergence tolerance with a yield advantage of 2–3.5 t ha^−1^ in farmers’ fields (Singh et al. 2009). However, these varieties perform poorly under stagnant flooding conditions, especially since most of the Sub1 varieties are relatively short, making the plants more prone to damage if water depth stays up to canopy level for longer than 2 weeks (Singh et al. 2011).

Conventional breeding to develop improved varieties with stagnant flooding has long been conducted at IRRI, and some tolerant lines have been developed (Collard et al. 2013; Mackill et al. 2010). Thus far, however, studies on genetic and molecular basis of stagnant flooding had not been initiated. In this study we report for the first time, the identification of QTLs under stagnant flooding conditions from an F_7_ RIL population derived from a cross of the improved version of the popular high-yielding Indonesian variety, Ciherang-Sub1 (IR09F436) (Septiningsih et al. 2015) and an IRRI submergence and stagnant flooding tolerant breeding line, IR10F365 (Collard et al. 2013) using an Illumina 6 K SNP chip platform (Thomson 2014). A subset of this RIL population was recently used in our study to identify non-Sub1 QTLs for tolerance to submergence during vegetative stage (Gonzaga et al. 2017). In the current study, 15 traits related to yield and important agronomic traits were investigated in both stagnant flooding and normal irrigated field conditions. The parallel study under control irrigated conditions is also valuable since the RIL population was developed using a popular cultivar (Ciherang-Sub1) and an elite line (IR10F365) which are both high yielding lines. Additionally, it allows detecting QTLs associated with responses induced by stagnant flooding versus inherent responses, with the former being most important for breeding varieties adapted to stagnant flooding conditions.

## Methods

### Plant material and development of the mapping population

A RIL mapping population was developed comprising of 156 F_7_ lines derived from a cross of Ciherang-Sub1 with IR10F365. Both parents carry the tolerant allele of the *SUB1* gene (i.e. Sub1 gene was fixed in this population), which contributes to tolerance to submergence during vegetative stage. A subset of 115 RILs from this population was used to map non-Sub1 QTLs for submergence tolerance during vegetative stage (Gonzaga et al. 2017); however, in this current study the entire population of 156 RILs was used. During the final analysis, however, 8 lines were removed due to high levels of missing genotype or phenotype data, resulting in a final set of 148 RILs. Other than the two parents, Swarna-Sub1 and IRRI154 were also included as susceptible and tolerant checks, respectively. IRRI154 (also known as NSIC Rc222) is a popular high-yielding irrigated rice variety released in the Philippines. This variety is highly tolerant to stagnant flooding but susceptible to complete submergence during vegetative stage. Swarna-Sub1 is the first submergence tolerant variety released in South Asia (Neeraja et al. 2007).

### Experimental field conditions

The mapping population was evaluated in 2014 wet season (WS) in the field plots at the International Rice Research Institute (IRRI), Los Baños, Laguna, Philippines (14°10’11.81”N, 121°15’39.22”E) under stagnant flooding stress and normal irrigated conditions. A row-column design was used with two replications, with planting distance of 20 × 20 cm in individual plots of 5 × 0.8 m under stress condition. The same population was planted under controlled irrigation using the same experimental design and planting conditions following standard agronomic practices. Besides the two parents, IRRI154 and Swarna-Sub1 were also included as checks. Fertilizer was applied at three stages as recommended at basal, maximum tillering and panicle initiation. Seeds were sown in raised seed bed nursery and 16 d-old seedlings were transplanted in the submergence plot with one seedling per hill. The water in the SF plot was raised to 10 cm at 2 weeks after transplanting (WAT) and was gradually increased by 5 cm each week until the 6^th^ WAT. Thereafter, water was raised gradually by 10 cm until the 8^th^ WAT, for a final depth of 50 cm. During this period plants were at maximum tillering stage, and this condition was maintained up to harvesting time. Water depth in the control field was kept at about 5 cm through maturity.

### Phenotyping and data analysis

Sixteen traits selected based on previous physiological studies of stagnant flooding tolerance or related studies were investigated (Hattori et al. 2007; Hattori et al. 2008; Kato et al. 2014; Nagai et al. 2014; Singh et al. 2011; Vergara et al. 2014). Those traits were: days to flowering (DTF), plant height (PH), shoot elongation rate (SER), number of tillers (TN), number of panicles (PN), 100 grain weight (GW), shoot biomass (BM), flag leaf length (FLL), flag leaf width (FLW), panicle length (PL), harvest index (HI), leaf sheath length for the first, second and third internodes (LSL_1_, LSL_2_, and LSL_3_, respectively), grain yield per plot (GY), and survival rate (SR). The same traits were measured under irrigated condition for direct comparisons, except SR. Details of phenotyping are provided in Additional file 1: Table S1. Data were analyzed using PB Tools 1.4 and STAR (http://bbi.irri.org/products). Analysis of variance (ANOVA) and correlation analysis were performed. Means were estimated using linear mixed models and broad-sense heritability (H^2^) was calculated using PBTools.

### Genotyping

Total genomic DNA was extracted using a modified CTAB technique (Murray and Thompson 1980), and the final concentration was normalized to 50 ng/μL. Genotyping was performed using the Illumina Infinium genotyping platform with a 6 K SNP chip designed by Susan McCouch at Cornell University and run at the Genotyping Services Laboratory, IRRI (Thomson 2014), as described previously (Gonzaga et al. 2017).

### QTL analysis and linkage map construction

Map Manager QTX, vQTXb20 (Manly et al. 2001) was used for linkage map construction with Kosambi map function. Due to high rates of missing data in 8 lines, only 148 RILs were used in the final analysis. QTL analysis was performed using QTL Cartographer v2.5 (Wang et al. 2010) with 1,000 permutations in both interval mapping (IM) and composite interval mapping (CIM) methods to determine the threshold at *P* ≤ 0.05 and *P* ≤ 0.01 to declare the significance of QTLs. Forward-backward stepwise regression of F-in = 0.01 and F-out = 0.01 was used in CIM. For comparison, QGene (Nelson 1997) was also used based on IM and CIM with permutation of 10,000 iterations. Standard rice QTL nomenclature was used (McCouch 2008).

### QTL comparison

QTLs identified in this study were compared with similar QTLs identified in previous studies using the Gramene QTL database (http://archive.gramene.org/qtl/) and QTL Annotation Rice Online (Q-TARO) database (http://qtaro.abr.affrc.go.jp/) (Yonemaru et al. 2010). QTLs for the same traits sharing similar regions between the irrigated and stagnant flooding environments and clusters of QTLs governing various traits located in similar regions are also reported.

## Results and Discussion

### Performance of parents and checks

*SUB1A* suppresses shoot elongation during complete submergence to limit carbohydrate consumption and increase chances of survival after water recedes; but this can lead to a disadvantage if submergence is followed by a longer duration of stagnant flooding (Sarkar et al. 2009; Singh et al. 2009). It was also reported that the first six Sub1 lines developed by IRRI (Septiningsih et al. 2009) were susceptible to stagnant flooding (Vergara et al. 2014). The susceptibility of Sub1 lines was worse with varieties having short stature, as in the case of Swarna-Sub1—which was used as a susceptible check in our stagnant flooding stress phenotyping, showing very low survival by the end of the experiment. On the other hand, the tolerant check IRRI154 performed well, confirming our previous results (Kato et al. 2014). There is no significant difference of survival rates among Ciherang-Sub1 (69%), IRRI154 (62%), and IR10F365 (56%) (Table 1). The grain yield under stagnant flooding was also higher for IRRI154 (3,394 kg ha^−1^) and Ciherang-Sub1 (2,933 kg ha^−1^), but significantly lower for IR10F365 (1,246 kg ha^−1^) and much lower for Swarna-Sub1 (154 kg ha^−1^). The yield reduction of Ciherang-Sub1 under SF compared to the control was comparable to that of IRRI154 (49.5 vs. 51.3%). In addition, Ciherang-Sub1 matured 5 days earlier under non-stress and 8 days earlier under stress compared with IRRI154 and IR10F365 (Tables 1 and 2). Under non-stress conditions, Ciherang-Sub1 had similar numbers of tillers and panicles compared to IRRI154 and IR10F365; while Swarna-Sub1 had a higher number of tillers and panicles. However, under stress tiller number and panicles of both Ciherang-Sub1 and IR10F365 was significantly reduced compared to IRRI154. Likewise, Ciherang-Sub1 and IR10F365 biomass was significantly affected under the stress (reduced by 40.9 and 40.7%, respectively), while IRRI154 biomass was hardly affected and Swarna-Sub1 was severely affected (reduced by 71.1%). It was also observed that IRRI154 had the highest shoot elongation rate under stress among the four varieties used in this study. The trend was also demonstrated in our earlier finding in which IRRI154 had the least biomass reduction and the fastest elongation compared with the other four varieties used in the study, including another tolerant check, IRRI119 or PSB Rc68 (Kato et al. 2014).

**Table 1:** Descriptive statistics of each trait in the mapping population, parents, and checks under stagnant flooding

Traits^a)^	Ciherang-Sub1	IR10F365	IRRI154	Swarna-Sub1	Population Mean	Highest line	Lowest line	LSD .05	H^2b)^	*P* ^c)^
DTF (d)	96	104	104	117	96	107	88	4.20	0.88	***
PH (cm)	122.8	118.2	123.9	57.0	129.3	154.7	94.6	19.00	0.62	***
TN	5	7	10	4	7	11	4	3.40	0.21	ns
PN	5	6	10	4	6	11	4	3.20	0.33	**
FLL (cm)	27.5	30.8	40.4	12.7	29.6	38.6	22.0	7.40	0.5	***
FLW (cm)	1.9	1.6	1.8	0.9	1.8	2.1	1.5	0.28	0.4	***
PL (cm)	25.2	27.6	25.8	12.2	25.5	29.7	20.0	4.26	0.45	***
BM (g/m^2^)	768.5	853.7	1230.8	520.5	985.0	1544.7	513.2	527.80	0.18	ns
SER (cm/d)	1.2	1.2	1.4	0.6	1.3	1.8	0.8	0.28	0.73	***
HI	0.36	0.14	0.29	0.01	0.28	0.56	0.10	0.20	0.39	**
GW (g)	2.8	2.5	2.4	0.7	2.7	3.2	1.6	0.28	0.85	***
LSL_1_ (cm)	12.6	13.8	11.0	3.2	14.2	24.4	9.2	4.40	0.45	***
LSL_2_ (cm)	15.2	17.8	13.1	6.4	17.4	24.0	12.0	5.60	0.45	**
LSL_3_ (cm)	29.2	21.7	29.7	13.4	27.9	35.4	17.8	7.40	0.43	***
GY (kg/Ha)	2933	1246	3394	154	2685	5184	49	1543.80	0.64	***
SR (%)	69	56	62	1	68	89	40	27.40	0.22	*

^a)^ *DTF* days to flowering, *PH* plant height, *TN* number of tillers, *PN* number of panicles, *FLL* flag leaf length, *FLW* flag leaf width, *PL* panicle length, *BM* shoot biomass, *SER* shoot elongation rate, *HI* harvest index, *GW* 100 grain weight, *LSL*_*1*_ *, LSL*_*2*_ *, and LSL*_*3*_ *, respectively* leaf sheath length for the first, second and third internodes, *GY* grain yield per plot, and *SR* survival rate

^b)^Heritability

^c)^ns: non significant; *significant at *P* ≤ 0.05; **significant at *P* ≤ 0.01; ***significant at *P* ≤ 0.001

**Table 2:** Descriptive statistics of each trait in the mapping population, parents, and checks under irrigated control condition

Traits^a)^	Ciherang-Sub1	IR10F365	IRRI154	Swarna-Sub1	Population Mean	Highest line	Lowest line	LSD .05	H^2b)^	*P* ^c)^
DTF (d)	87	92	92	106	89	96	80	2.81	0.95	***
PH (cm)	118.1	122.8	112.8	109.0	125.3	139.3	99.92	8.32	0.88	***
TN	10	11	12	16	10	13	7	2.68	0.54	***
PN	10	11	11	16	10	18	7	3.41	0.32	*
FLL (cm)	29.5	38.0	36.4	26.3	32.0	43.09	23.91	6.57	0.61	***
FLW (cm)	2.1	1.8	1.7	1.9	1.9	2.35	1.54	0.31	0.53	***
PL (cm)	25.01	25.99	23.46	23.49	26.09	30.5	21.54	2.22	0.78	***
BM (g/m^2^)	1301.33	1438.67	1262.24	1803.67	1329.34	1762.8	757.2	399	0.25	*
SER (cm/d)	1.4	1.1	1.3	1.0	1.3	1.8	0.9	0.31	0.55	***
HI	0.45	0.31	0.56	0.18	0.43	0.6	0.22	0.16	0.45	**
GW (g)	2.7	2.4	2.4	2.0	2.7	3.12	2.39	0.17	0.89	***
LSL_1_ (cm)	9.8	14.0	11.9	11.0	14.1	23.96	5.65	4.73	0.55	***
LSL_2_ (cm)	20.3	20.0	18.4	17.0	22.1	28.1	12.6	4.82	0.53	***
LSL_3_ (cm)	35.1	30.9	31.1	27.3	32.7	41.16	25	5.08	0.74	***
GY (kg/Ha)	5809	4680	6974	3391	5605	7398	3600	1322.80	0.59	***

^a)^ *DTF* days to flowering, *PH* plant height, *TN* number of tillers, *PN* number of panicles, *FLL* flag leaf length, *FLW* flag leaf width, *PL* panicle length, *BM* shoot biomass, *SER* shoot elongation rate, *HI* harvest index, *GW* 100 grain weight, *LSL*_*1*_ *, LSL*_*2*_ *, and LSL*_*3*_ *, respectively* leaf sheath length for the first, second and third internodes, and *GY* grain yield per plot

^b)^Heritability

^c)^ns: non significant; *significant at *P* ≤ 0.05; **significant at *P* ≤ 0.01; ***significant at *P* ≤ 0.001

The current study showed that even though Ciherang-Sub1 elongation was less than that of IRRI154 (4.0 vs. 9.8%) and its biomass reduction was more affected compared to IRRI154, Ciherang-Sub1 had better tolerance to stagnant flooding compared to the first generation of Sub1 lines, which were the first six Sub1 lines developed by IRRI (Vergara et al. 2014). The good performance of Ciherang-Sub1 could partly be the result of its intermediate height (118.1 cm), which was similar to IR10F365 (122.8 cm), slightly taller than IRRI154 (112.8 cm) and significantly taller than Swarna-Sub1 (106 cm) under control condition. While under stagnant flooding its height (122.8 cm) was similar to IRRI154 (123.9 cm) and IR10F365 (118.2 cm), but significantly taller than that of Swarna-Sub1 (57 cm). Shoot elongation rate of Ciherang-Sub1 (1.2 cm/d) was similar to IR10F365 (1.2 cm/d) and IRRI154 (1.4 cm/d), but significantly faster than that of Swarna-Sub1 (0.6 cm/d). As shown in our previous study, IRRI154 tolerance was mostly due to its medium elongation rate, which enabled its canopy to keep up with the water surface (Kato et al. 2014). In summary, the advantage of Ciherang-Sub1 under stagnant flooding is its partial elongation and inherent intermediate height.

IR10F365, a breeding line from IRRI’s submergence breeding team, has consistently shown strong submergence and stagnant flooding tolerance in field trials. This line has also performed well in trials from the National Coordinated Trials of the Philippines in flood-prone areas. In our current study, however, IR10F365 slightly underperformed compared to Ciherang-Sub1 in both control and stagnant flooding. This might be due to the use of older seed stock of this particular line (due to unavailability of fresh seeds from the same batch) which may have caused a slower rate of germination and growth, which subsequently affected yield. For our mapping population, however, other than the parental lines we also included the susceptible check Swarna-Sub1 and the tolerant check IRRI154 which can also be used as negative and positive checks, respectively, when we investigate the performance of the selected individuals in the mapping population.

### Mapping population performance under stagnant flooding vs. control conditions

The mean grain yield (GY) of the population decreased significantly, by 52.1% under SF (Tables 1 and 2). Kato et al. (2014) reported similar results where yield under stress was reduced by 47% across genotypes. The best performing line under SF (RIL-214) yielded 5,184 kg ha^−1^ with a yield advantage of 43.5% over Ciherang-Sub1 and 34.5% over the best tolerant check, IRRI154. The large yield increase under stress, however, was not reflected under irrigated conditions since the yield of the same was 5,676 kg ha^−1^ under control conditions compared to the most productive line (RIL-186) at 7,398 kg ha^−1^ (Additional file 2: Table S2). However, RIL-186 yield was only 2,584 kg ha^−1^ under SF, suggesting that this line is less tolerant. Interestingly, the sixth best line under SF was also at the same rank under control conditions, with grain yield of 4,067 and 6,681 kg ha^−1^, respectively. Therefore, it is possible to develop high-yielding lines that thrive in both environments out of this population, even though they might not be the best performers in either environment. Likewise, the tolerant check IRRI154 performs well in both control and SF conditions, but the downside of this variety is its low grain quality and sensitivity to complete submergence. The worst performer under SF only yielded 921 kg ha^−1^. Compared to this line, the best performer under stress was 24.0% taller, had more than double its biomass, with 44% faster rate of shoot elongation and had more than triple harvest index. It was worth noting that under SF, 26 RILs yielded higher than IRRI154 and 60 RILs yielded higher than Ciherang-Sub1. Under control conditions, only one line has higher yield than IRRI154, while 66 lines were better than Ciherang-Sub1 (data not shown). For 100 grain weight (GW), the mean of the population in both conditions was unchanged (Tables 1 and 2). The averages biomass (BM) and harvest index (HI) of the population were reduced by 25.9 and 34.9% under stress, respectively. Among these traits, heritability (H^2^) was highest for grain weight in both stress and control conditions (0.85 vs. 0.89), while heritability for grain yield was moderate (0.64 vs. 0.59), and low for HI and BM.

The mean of plant height (PH) under SF similar is similar compared to the control (only 3.4% increased); while the mean shoot elongation rate (SER) stayed the same under both environments (Tables 1 and 2). Under SF, mean days to flowering (DTF) was 96 days, which was equal to that of Ciherang-Sub1 but earlier than IR10F365 and IRRI154 by 8 days and Swarna-Sub1 by 21 days. The mean of DTF under control condition was 89 days, which was not significantly difference compared to those of Ciherang-Sub1, IR10F365 and IRRI154, but 17 days earlier than Swarna-Sub1. The overall mean delay caused by SF across the mapping population was 7 days. It was previously reported that stagnant flooding delays flowering.

Under SF, tillers and panicle number were reduced by 30 and 40%, respectively, compared to the control, which was similar to that of IR10F365 and Ciherang-Sub1 (Tables 1 and 2). Interestingly, the average panicle length of the mapping population, along with those of Ciherang-Sub1, IR10F365, and IRRI154 were hardly affected. Likewise, the population averages of flag leaf length (FLL) and flag leaf width (FLW) were only slightly reduced. Average leaf sheath length for the 1^st^ internode (LSL_1_) of the mapping population was less affected; however, average leaf sheath length for the 2^nd^ and 3^rd^ internodes (LSL_2_ and LSL_3_) of the population were reduced by 21.3 and 15.3%, respectively. This trend was similar to those of the parents and checks, with Swarna-Sub1 being the most affected. Overall, the population has high mean of survival rate under SF, ranging from 40 to 89%, with an average of 68%, which was similar to that of Ciherang-Sub1 (69%).

To investigate the effect of individual traits on yield stability across stagnant flooding and irrigated conditions, the percentage difference for each trait was calculated for the 25 highest and 25 lowest yielding lines under SF and the control (Additional file 3: Figure S1 and Additional file 4: Figure S2). Under SF, the two traits with biggest differences were GY and HI; this was followed by moderate differences in PN, BM, SER, SR, all LSL, TN, PH, and FLL. These traits largely contribute to higher HI and yield. Under control condition, the two largest contrasting traits were also GY and HI, even though the differences were smaller compared to those under stress. It was also shown that the lines with higher shoot elongation rate had higher survival rate but not all performed well. The lines having elongation rate between 1.2 and 1.6% were high yielding and had good survival rate of 60–80% (Additional file 5: Figure S3). This was likely due to high carbohydrate consumption in fast-elongating lines to avoid complete submergence, but at the expense of yield. However, moderate SER was strongly and positively correlated with grain yield under stress, as previously reported (Kato et al. 2014; Vergara et al. 2014).

### Correlation among traits

Under stagnant flooding, grain yield of the mapping population correlated positively with all the traits, except DTF. Traits having very highly significant correlation (***; *p*-value ≤ 0.001) with GY were HI (0.70), PH (0.58), SER (0.44), BM (0.43), LSL_3_ (0.43), FLW (0.40) and LSL_1_ (0.40), LSL_2_ (0.40), PN (0.36), PL (0.31), FLL (0.29), and GW (0.29); while TN (0.25) had highly significant correlation (**; *p*-value ≤ 0.01; Additional file 6: Table S3). Survival rate was correlated with all the traits, except HI. Traits having very highly significant correlation with SR were PH (0.61), BM (0.47), GW (0.47), LSL_2_ (0.43), PL (0.42), FLW (0.41), LSL_1_ (0.40), LSL_3_ (0.39), SER (0.39), PN (0.38), and FLL (0.30); while traits having highly significant correlation were TN (0.25) and DTF (−0.24). All LSS traits were positively very highly significant correlation with PH and SER, but negatively very highly significant correlation with DTF (Additional file 6: Table S3). Under SF, all traits were correlated with plant height. Traits having very highly significant correlation with PH were LSL_2_ (0.72), BM (0.67), LSL_1_(0.63), SER (0.62), SR (0.61), GW (0.59), PL (0.59), GY (0.58), LSL_3_ (0.55), FLW (0.54), FLL (0.37), and PN (0.28); while traits having highly significant correlation were TN (0.24) and DTF (−0.24), and trait having significant correlation (*; *p*-value ≤ 0.05) was HI (0.18). Additionally, DTF were negatively very highly significant correlation with SER (−0.56), LSL_1–3_ (−0.41, −0.41, −0.42, respectively), and FLL (−0.33); while it was negatively highly significant correlated with SR (−0.24) and PH (−0.24), and negatively significant correlated with HI (−0.21), FLW (−0.21) and TN (−0.16).

In general, the correlations among traits were weaker under control conditions than under SF. Under control conditions, the only trait that was very highly significant correlated (***) with GY was HI (0.71); it was also highly significantly correlated (**) with LSL_2_ (0.25) and DTF (−0.25), and significantly correlated (*) with SER (0.17) (Additional file 7: Table S4). All LSL were very highly significantly correlated with PH. Under control conditions, DTF had either positive or negative correlation with some of the traits (Additional file 7: Table S4).

### Identification of QTLs

**Fig. 1:**
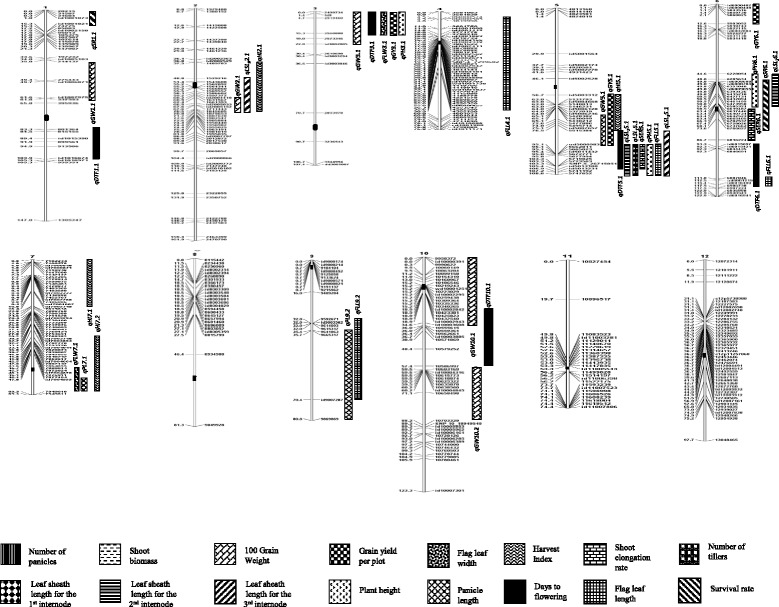
Linkage map of a RIL population (F_7_) derived from a cross between Ciherang-Sub1 and IR10F365 under stagnant flooding along with the positions of QTLs for 16 traits. QTLs identified are indicated above each bar, traits are indicated by the patterns of the bars. Centromeres are shown as *black* boxes in each chromosome

**Fig. 2:**
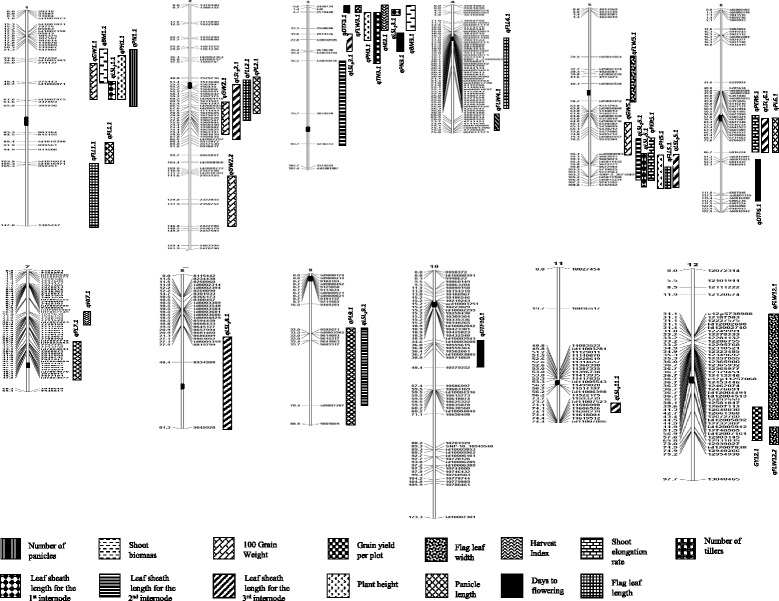
Linkage map of a RIL population (F_7_) population derived from a cross between Ciherang-Sub1 and IR10F365 under control irrigated condition along with the positions of QTLs for 15 traits. The QTL boundaries are indicated by the closest flanking markers, traits are indicated by the patterns of the bars. Centromeres are shown as *black* boxes in each chromosome

Out of 4606 high quality SNP markers on the 6 K SNP chip, 10% (469) were polymorphic between Ciherang-Sub1 and IR10F365. The rice physical map of Nipponbare (MSU v.7) was used to order the markers. The marker distances were calculated from the genotype data using MapManager QTX vQTXb20 (Manly et al. 2001) and had a total length of 1,250.4 cM with an average of 2.74 cM between markers. QTL analysis using both QTL Cartographer and QGene identified a total of 38 and 46 QTLs under stagnant flooding and the control, respectively (Tables 3 and 4; Figs. 1 and 2). There were 16 QTLs detected in both environments; in addition several QTL clusters were observed. Under SF, QTLs were detected from 13 out of 16 traits (except PN, TN, and BM), while under control, QTLs were detected from all 15 traits. Most of the QTLs were in similar positions as previously reported QTLs (Tables 5 and 6).

**Table 3:** QTLs for yield in agronomic traits from a RIL population of Ciherang-Sub1/IR10F365 identified under stagnant flooding condition

Trait^a)^	QTL	Chr	Flanking markers	Source^b)^	QTL Cart. CIM			QTL Cart. IM^c)^			QGENE CIM			QGENE IM		
					LOD	R^2^	Add	LOD	R^2^	Add	LOD	R^2^	Add	LOD	R^2^	Add
GW	*q GW1.1*	1	214137-id1007975	C	**4.36**	**8.7**	**−0.06**	**4.21**	**12.3**	**−0.07**	**3.35**	**10.0**	**0.05**	**3.24**	**10.0**	**0.07**
	*q GW2.1*	2	id2004418-1845605	C				**2.70**	**8.0**	**−0.05**						
	*q GW3.1*	3	2499734-id3002805	C				**2.80**	**10.2**	**−0.06**						
	*qGW5.1*	5	5428382-ud5000983	C	**4.07**	**8.3**	**−0.06**	**3.80**	**11.0**	**−0.06**	**3.73**	**11.0**	**0.08**	**4.17**	**12.0**	**0.07**
	*qGW10.1*	10	10603169-10703329	I	**6.30**	**13.1**	**0.07**	**3.15**	**10.0**	**0.06**						
	*qGW10.2*	10	9958372-id10003608	C	**4.14**	**9.0**	**−0.06**				**3.41**	**10.0**	**−0.07**	*2.50*	*8.0*	*−0.06*
DTF	*qDTF1.1*	1	801364-id1016674	I	**3.08**	**6.0**	**0.88**	*2.78*	*8.2*	*1.03*	*3.40*	*10.0*	*−0.89*			
	*qDTF3.1*	3	2499734-2560888	C	**12.50**	**26.0**	**−1.80**	**6.81**	**20.0**	**−1.57**	**5.43**	**16.0**	**1.20**	**6.00**	**17.0**	**1.49**
	*qDTF5.1*	5	5515384-id5013231	C	**4.11**	**7.0**	**−1.01**	**3.17**	**9.3**	**−1.13**	**3.65**	**11.0**	**1.27**	**3.63**	**11.0**	**1.23**
	*qDTF6.1*	6	id6015097-6906738	I				**3.95**	**12.0**	**1.54**				**4.37**	**13.0**	**−1.73**
	*qDTF10.1*	10	id10002842-10586997	I	**5.22**	**9.0**	**1.07**	**3.01**	**9.0**	**1.04**	*2.68*	*8.0*	*−0.82*			
FLW	*qFLW3.1*	3	2499734-2560888	C	**4.00**	**11.3**	**−0.04**	**3.47**	**11.0**	**−0.04**				**3.22**	**10.0**	**0.04**
	*qFLW7.1*	7	7869914-7949610	C				**3.11**	**14.0**	**−0.04**						
FLL	*qFLL4.1*	4	*id4000574-4295290*	I	**4.11**	**9.3**	**1.10**	**2.78**	**8.2**	**1.03**	**3.79**	**11.1**	**−1.12**	**2.87**	**8.5**	**−1.08**
	*qFLL5.1*	5	ud5000983-5747652	I	**6.18**	**14.0**	**1.36**	**3.69**	**11.0**	**1.17**				**3.53**	**10.4**	**−1.18**
	*qFLL6.1*	6	6887046-6906738	C				*2.71*	*8.5*	*−1.28*				**2.98**	**8.9**	**1.42**
	*qFLL9.1*	9	9592671-id9007287	I	**2.94**	**13.0**	**1.23**				**2.99**	**8.9**	**−1.65**	*2.60*	*7.8*	*−1.85*
GY	*qGY3.1*	3	2499734-2560888	C	**6.20**	**14.3**	**−321.60**	*2.83*	*9.0*	*−246.10*	*2.64*	*8.0*	*224.3*	**2.84**	**8.4**	**250.46**
	*qGY5.1*	5	id5003312-ud5000983	I	**3.66**	**12.2**	**301.7**				*2.15*	*7.0*	*−190.7*			
	*qGY6.1*	6	id6000402-5903052	C	**3.46**	**7.4**	**−282.1**				**3.04**	**9.0**	**263.8**			
HI	*qHI2.1*	2	1489783-1845605	I	**3.41**	**72.4**	**0.03**				*2.18*	*6.6*	*−0.02*			
	*qHI5.1*	5	id5003312-5515384	I	**3.34**	**72**	**0.03**									
	*qHI7.1*	7	7768382-7949610	C	**8.67**	**20.4**	**−0.05**	**3.64**	**10.6**	**−0.03**	**7.07**	**19.7**	**0.05**	**3.80**	**11.1**	**0.03**
	*qHI7.2*	7	7102234-id7002749	I	**4.65**	**10**	**0.03**									
LSL_1_	*qLSL*_*1*_ *5.1*	5	ud5000983-5747652	I	**4.40**	**11.0**	**0.74**	**4.00**	**12.0**	**0.76**				**4.04**	**12.0**	**−0.79**
LSL_2_	*qLSL*_*2*_ *5.1*	5	ud5000983-5747652	I	**4.73**	**10.2**	**0.82**	**4.86**	**15.0**	**0.96**	*2.57*	*7.7*	*−0.82*	**4.96**	**14.3**	**−0.98**
	*qLSL*_*2*_ *6.1*	6	6228054-6585321	I	**4.10**	**9.0**	**0.91**							**3.66**	**11.0**	**−1.38**
LSL_3_	*qLSL*_*3*_ *2.1*	2	1529216-1845605	C	**4.16**	**10.0**	**−1.06**	**2.99**	**9.0**	**−1.00**	*2.37*	*7.1*	*0.85*	**2.78**	**8.3**	**0.97**
	*q LSL*_*3*_ *5.1*	5	5515384-5747652	I	**3.49**	**8.0**	**1.00**	*2.72*	*9.0*	*1.04*				**3.17**	**9.4**	**1.11**
SER	*qSER5.1*	5	ud5000983-id5013231	I	**12.5**	**32.0**	**0.11**	**8.63**	**24.0**	**0.09**	**4.12**	**12.0**	**−0.06**	**9.20**	**24.9**	**−0.10**
	*qSER6.1*	6	6619487-6816224	C	**3.05**	**6.0**	**−0.05**	*2.82*	*8.3*	*−0.06*	**3.79**	**11.1**	**0.05**	**2.87**	**8.5**	**0.06**
PH	*qPH3.1*	3	2499734-2560888	C	**4.04**	**8.3**	**−2.77**	**3.10**	**9.4**	**−2.88**	*3.34*	*10.0*	*2.67*	*2.57*	*8.00*	*2.69*
	*qPH5.1*	5	ud5000983-5747652	I	**5.59**	**12.1**	**3.45**	**4.73**	**14.0**	**3.65**				**4.97**	**14.3**	**−3.81**
	*qPH6.1*	6	6228054-6585321	I							**3.40**	**10.0**	**−3.74**			
PL	*qPL7.1*	7	id7004922-7949610	I	**3.21**	**11.2**	**0.61**									
	*qPL9.1*	9	9641863-9869869	I	**5.47**	**22.0**	**0.82**	**4.42**	**21.2**	**0.81**	*3.29*	*10.0*	*−0.53*	**3.64**	**11.0**	**−0.61**
SR	*qSR1.1*	1	20215-id1001821	I	**2.89**	**10.0**	**3.18**				*2.63*	*8.0*	*−2.77*			
	*qSR6.1*	6	id6008688-6766627	C	**2.97**	**7.3**	**−2.99**				*2.50*	*7.4*	*4.01*			

^a)^ *DTF* days to flowering, *FLW* flag leaf width, *FLL* flag leaf length, *GY* grain yield per plot, *HI* harvest index, *LSL*_*1*_ *, LSL*_*2*_ *, and LSL*_*3*_ *, respectively* leaf sheath length for the first, second and third internodes, *SER* shoot elongation rate, *PH* plant height, *PL* panicle length, and *SR* survival rate

^b)^C: Ciherang-Sub1; I: IR10F365

c)underlined and bold numbers: significant at *P* ≤ 0.01; bold numbers: significant at *P* ≤ 0.05; italic: not significant but with LOD ≥ 2.0

**Table 4:** QTLs for yield in agronomic traits from a RIL population of Ciherang-Sub1/IR10F365 identified under irrigated control condition

Trait	QTL	Chr	Flanking markers	Source	QTL Cart. CIM			QTL Cart. IM			QGENE CIM			QGENE IM		
					LOD	R^2^	Add	LOD	R^2^	Add	LOD	R^2^	Add	LOD	R^2^	Add
GW	*qGW1.1*	1	214137-id1007975	C	**4.74**	**11.4**	**−0.05**	**5.34**	**15.2**	**−0.05**	**4.40**	**12.8**	**0.06**	**4.35**	**12.7**	**0.05**
	*qGW2.1*	2	1661393-1981019	C	**3.29**	**6.5**	**−0.04**	**4.21**	**12.2**	**−0.05**	**3.13**	**9.3**	**0.04**	**3.14**	**9.3**	**0.04**
	*qGW2.1*	2	2153125-2422788	C							**2.74**	**8.2**	**−0.04**			
	*qGW5.1*	5	5428382-ud5000983	C	*2.39*	*5.0*	*−0.03*	**3.60**	**10.5**	**−0.04**	*2.51*	*7.5*	*0.04*	**3.13**	**9.3**	**0.04**
DTF	*qDTF3.1*	3	2499734-2519460	C	**21.81**	**42.2**	**−2.44**	**13.53**	**34.9**	**−2.20**	**17.36**	**41.7**	**2.35**	**13.14**	**33.6**	**2.22**
	*qDTF6.1*	6	id6015421-6906738	I				**4.09**	**11.9**	**1.70**				**3.70**	**10.9**	**−1.70**
	*qDTF10.1*	10	id10003608-10579252	I	**5.70**	**9.0**	**1.13**	**2.99**	**8.8**	**1.11**	**5.43**	**15.5**	**−1.18**			
FLW	*qFLW3.1*	3	2499734-2519460	C	**5.18**	**11.0**	**−0.05**				**3.49**	**10.3**	**0.10**			
	*qFLW4.1*	4	4683923-4728960	C	**8.88**	**20.1**	**−0.09**	**3.73**	**10.9**	**−0.06**	**3.98**	**11.6**	**0.06**	**3.29**	**9.7**	**0.06**
	*qFLW5.1*	5	id5001564-id5003312	C	*2.67*	*5.4*	*−0.04*							**2.72**	**8.1**	**−0.05**
	*qFLW12.1*	12	c12p5738988-id12005832	I	**4.80**	**10.3**	**0.06**									
	*qFLW12.2*	12	12740505-12939027	C	**3.44**	**7.3**	**−0.05**									
FLL	*qFLL1.1*	1	id1016971-1305247	C	**3.81**	**25.2**	**−1.79**									
	*qFLL2.1*	2	1543608-1902547	I	**3.00**	**6.8**	**0.96**	*2.97*	*8.8*	*1.06*	**3.85**	**11.3**	**−1.19**	**3.54**	**10.4**	**−1.17**
	*qFLL4.1*	4	id4002348-4253547	I	**3.05**	**6.8**	**0.99**				**3.15**	**9.3**	**−1.15**			
	*qFLL5.1*	5	5719825-5747652	I	**3.90**	**9.1**	**1.12**									
GY	*qGY12.1*	12	12648838-12931835	I	**3.39**	**9.2**	**247.20**				*2.27*	*6.8*	*−206.30*			
HI	*qHI3.1*	3	2499734-2560888	I	**3.50**	**8.0**	**0.02**				**4.91**	**14.2**	**−0.03**	*2.25*	*6.7*	*−0.02*
	*qHI7.1*	7	id7002749-7730100	C							**3.50**	**10.3**	**0.05**			
LSL_1_	*qLSL*_*1*_ *1.1*	1	275443-id1007975	C	**4.37**	**9.8**	**−0.78**	**3.18**	**9.4**	**−0.77**						
	*qLSL*_*1*_ *3.1*	3	fd9-2519460	C	**5.33**	**13.1**	**0.93**	**3.78**	**11.0**	**−0.82**	**3.95**	**11.6**	**0.83**	**3.23**	**9.6**	**0.76**
	*qLSL*_*1*_ *5.1*	5	ud5000983-5747652	I				**3.93**	**11.8**	**0.88**	**2.79**	**8.3**	**−0.86**	**3.80**	**11.1**	**0.88**
LSL_2_	*qLSL*_*2*_ *3.1*	3	id3003846-3236543	C	**3.70**	**8.1**	**−0.67**	*2.59*	*7.8*	*−0.65*	**3.23**	**9.6**	**0.94**			
	*qLSL*_*2*_ *5.1*	5	5515384-id5013231	I	**4.95**	**11.2**	**0.84**	*2.77*	*9.4*	*0.76*				**3.03**	**9.0**	**−0.83**
	*qLSL*_*2*_ *9.1*	9	9592671-id9007287	I	**4.33**	**16.8**	**0.97**	*2.84*	*17.6*	*0.99*	**4.45**	**12.9**	**−1.59**	**3.29**	**9.7**	**−1.45**
LSL_3_	*qLSL*_*3*_ *2.1*	2	1562092-id2007797	C				**3.92**	**11.4**	**−1.06**	**4.41**	**12.8**	**1.10**	**3.89**	**11.4**	**1.08**
	*qLSL*_*3*_ *3.1*	3	2573346-2610698	C	*2.63*	*4.1*	*−0.69*	**3.60**	**10.5**	**−1.03**	**4.40**	**12.8**	**1.24**	**3.18**	**9.4**	**1.03**
	*qLSL*_*3*_ *5.1*	5	ud5000983-5747652	I	**6.73**	**12.8**	**1.17**	**2.96**	**10.2**	**1.03**				**3.12**	**9.3**	**−1.02**
	*qLSL*_*3*_ *6.1*	6	6619487-6816224	C	**5.83**	**10.4**	**−1.10**									
	*qLSL*_*3*_ *8.1*	8	8803052-9049928	I	**7.41**	**14.7**	**1.29**	**4.18**	**22.1**	**1.49**	**6.24**	**17.6**	**−2.47**	**5.59**	**16.0**	**−2.38**
	*qLSL*_*3*_ *11.1*	11	id11007523-11608239	C	**5.22**	**9.3**	**−0.95**	**3.40**	**10.0**	**−0.97**	**4.03**	**11.8**	**1.19**	**3.69**	**10.9**	**1.05**
SER	*qSER5.1*	5	5515384-id5013231	I	**8.10**	**20.0**	**0.08**	**6.53**	**20.0**	**0.08**	**3.02**	**9.0**	**−0.06**	**7.08**	**19.8**	**0.09**
	*qSER6.1*	6	6585321-6816224	C	**3.70**	**8.0**	**−0.05**									
PH	*qPH1.1*	1	197232-id1007975	C				**3.13**	**10.2**	**−2.38**				*2.55*	*7.6*	*2.09*
	*qPH3.1*	3	2519460-id3002805	C	**3.15**	**7.0**	**−1.96**				**3.12**	**9.3**	**2.31**			
	*qPH5.1*	5	ud5000983-5747652	I	**9.90**	**23.9**	**3.71**	**8.22**	**22.4**	**3.57**	**3.98**	**11.7**	**−2.99**	**7.62**	**21.1**	**−3.58**
TN	*qTN3.1*	3	2519460-id3003846	I	**3.31**	**9.3**	**0.41**	**3.85**	**11.7**	**0.45**	**3.64**	**10.7**	**−0.49**	**4.29**	**12.5**	**−0.49**
PL	*qPL1.1*	1	id1015390-id1016971	C	**3.51**	**6.7**	**−0.44**									
	*qPL2.1*	2	1529216-1845605	I	**7.11**	**14.3**	**0.66**	**4.66**	**13.4**	**0.60**	**4.80**	**13.9**	**−0.62**	**4.59**	**13.3**	**−0.61**
	*qPL6.1*	6	6619487-6816224	C	**4.91**	**10.3**	**−0.55**	**5.92**	**18.4**	**−0.72**	**5.01**	**14.4**	**0.66**	**4.81**	**13.9**	**0.64**
	*qPL7.1*	7	id7003853-id7004922	I				*2.84*	*8.4*	*0.49*	**3.29**	**9.7**	**−0.56**	**3.62**	**10.7**	**−0.58**
	*qPL9.1*	9	9592671-9869869	I				**3.36**	**20.4**	**0.73**	**3.27**	**9.7**	**−0.83**	**2.92**	**8.7**	**−0.73**
PN	*qPN1.1*	1	147907-395936	I				*2.55*	*7.6*	*0.39*	**3.49**	**10.3**	**−0.55**	**3.16**	**9.4**	**−0.55**
	*qPN3.1*	3	2573346-2610698	I				**2.88**	**8.9**	**0.43**	**2.74**	**8.2**	**−0.47**	**2.83**	**8.4**	**−0.44**
BM	*qBM1.1*	1	147907-275443	I	**3.92**	**10.2**	**51.73**	*2.44*	*72.0*	*43.16*	**3.68**	**10.8**	**−50.83**	*2.30*	*6.9*	*−42.4*
	*qBM3.1*	3	2499734-2560888	C	**2.89**	**7.0**	**−45.20**				**3.33**	**9.9**	**49.64**			

^a)^ *GW* 100 grain weight, *DTF* days to flowering, *FLW* flag leaf width, *FLL* flag leaf length, *GY* grain yield per plot, *HI* harvest index, *LSL*_*1*_ *, LSL*_*2*_ *, and LSL*_*3*_ *, respectively* leaf sheath length for the first, second and third internodes, *SER* shoot elongation rate, *PH* plant height, *TN* number of tillers, *PL* panicle length, *PN* number of panicles, and *BM* shoot biomass

^b)^C: Ciherang-Sub1; I: IR10F365

c)underlined and bold numbers: significant at *P* ≤ 0.01; bold numbers: significant at *P* ≤ 0.05; italic: not significant but with LOD ≥ 2.0

**Table 5:** Genomic positions of QTLs identified in stagnant flooding with its QTL cluster and comparison to the QTLs identified in control irrigated condition in this study and previously published QTLs

QTL	Studies shared common region
*qGY3.1*	(Xiao et al. 1996); *dth3.1*: (Moncada et al. 2001); *qtl3.1*: (Bernier et al. 2007); *qPH3.1, qFLW3.1, qGW3.1*: this study under SF; *qDTF3.1*: this study under SF and control
*qGY5.1*	*qYl-5*: (Cho et al. 2003); *qGW5.1*: this study under SF and control; *qHI5.1*: this study under SF
*qGY6.1*	*qDTY6.1:* (Venuprasad et al. 2012)
*qGW1.1*	*GW1-2*: (Yu et al. 2008); *qGW1.1*: this study under control
*qGW2.1*	*tgw2*: (Yoon et al. 2006); *qHI2.1, qLSL*_*3*_ *2.1*: this study under SF
*qGW3.1*	*QKw3a:* (Li et al. 1997); *qPH3.1, qFLW3.1, qGY3.1*: this study under SF; *qDTF3.1*: this study under SF and control
*qGW5.1*	*gw-5*: (Lu et al. 1997); *qGW5.1*: this study under control *qHI5.1* and *qGY5.1:* this study under SF
*qGW10.1*	*QKw10*: (Li et al. 1997)
*qGW10.2*	(Ishimaru 2003); *gw10.1*: (Thomson et al. 2003)
*qHI2.1*	*qGW2.1* and *qLSL*_*3*_ *2.1*: this study under SF
*qHI5.1*	*qGW5.1*: this study SF and control; *qGY5.1*: this study under SF
*qHI7.1*	none
*qHI7.2*	*qPL7.1* and *qFLW7.1*: this study under SF
*qSR1.1*	none
*qSR6.1*	*qSER6.1*: this study under SF and control
*qPL7.1*	(Mei et al. 2003); *qHI7.2, qFLW7.1*: this study under SF
*qPL9.1*	*qPL-9*: (Cho et al. 2003); *pl9.1*: (Septiningsih et al. 2003); *pl9.1*: (Thomson et al. 2003); (Marri et al. 2005); (Li et al. 2006); *qPL9.1*: this study under control
*qPH3.1*	(Li et al. 1997); *qPh3a*: (Li et al. 2003); *qGY3.1, qFLW3.1, qGW3.1*: this study under SF; *qDTF3.1*: this study under SF and control
*qPH5.1*	(Mei et al. 2003); *ph5.1*: (Moncada et al. 2001); *ph5.1*: (Thomson et al. 2003); *qPH5.1*: this study under control; *qLSL*_*1*_ *5.1, qLSI2-5.1, qLSI3-5.1, qSER5.1, qFLL5.1* and *qDTF5.1*: this study under SF and control
*qPH6.1*	*ph6*: (Xiao et al. 1996); *qPH2-6-1*: (Cui et al. 2004); *qLSL*_*2*_ *6.1*: this study under SF
*qSER5.1*	*qSER5.1*: this study under control; *qPH5.1, qLSL*_*1*_ *5.1, qLSI*_*2*_ *5.1, qLSI*_*3*_ *5.1, qSER5.1, qFLL5.1*, and *DTF5.1*: this study under SF and control
*qSER6.1*	*qSR6.1*: this study under SF and control
*qLSL*_*1*_ *5.1*	*qLSL*_*1*_ *5.1*: this study under control; *qPH5.1, qLSL*_*2*_ *5.1*; *qLSL*_*3*_ *5.1, qSER5.1, qFLL5.1*, and *DTF5.1*: this study under SF and control
*qLSL*_*2*_ *5.1*	*qLSL*_*2*_ *5.1*: this study undercontrol; *qPH5.1, qLSL*_*1*_ *5.1* and *qLSL*_*3*_ *5.1, qSER5.1, qFLL5*: this study under SF and control; *DTF5.1*: this study under SF
*qLSL*_*2*_ *6.1*	*qPH6.1*: this study under SF
*qLSL*_*3*_ *2.1*	*qHI2.1* and *qGW2.1*: this study under SF
*qLSL*_*3*_ *5.1*	*qLSL*_*3*_ *5.1*: this study under control; *qPH5.1, qLSL*_*1*_ *5.1*, *qLSL*_*2*_ *5.1, qSER5.1,* and *qFLL5.1*: this study under SF and control; *DTF5.1*: under SF
*qFLL4.1*	(Mei et al. 2005)
*qFLL5.1*	*QFll7*: (Bing et al. 2006); (Yan et al. 2003); *qFLL5.1*: this study under irrigated; *qPH5.1*, *qLSI1-5.1, qLSL*_*2*_ *5.1*, *qLSL*_*3*_ *5.1*, *qSER5.1*: this study under SF and control; *DTF5.1*: this study under SF
*qFLL6.1*	(Mei et al. 2005); *qDTF6.1*: this study under SF and control
*qFLL9.1*	*fll9*: (Yan et al. 1999)*; qPL9.1*: this study under SF and control
*qFLW3.1*	*qPH3.1, qGW3.1, qGY3.1*: this study under SF; *qDTF3.1*: this study SF and control
*qFLW7.1*	*qHI7.2, qPL7.1*: this study under SF
*qDTF1.1*	*qDTH-1*: (Cho et al. 2003); *QHd1b*: (Li et al. 2003); (Mei et al. 2003); *qFLL6.1* : this study under SF
*qDTF3.1*	(Albar et al. 1998); *dth3.1*: (Moncada et al. 2001); *qHD-3-1*: (Takeuchi et al. 2001); *qHDD3-1*: (Hittalmani et al. 2003); *QHd3a*: (Li et al. 2003); (Mei et al. 2003); *qEMF3*: (Hirabayashi et al. 2014); *qDTF3.1*: this study under control; *qGW3.1, qFLW3.1, qPH3.1, qGY3.1*: this study under SF
*qDTF5.1*	(Li et al. 1998); *QHd5b*: (Li et al. 2003); *qPH5.1*, *qLSL*_*1*_ *5.1, qLSL*_*2*_ *5.1*, *qLSL*_*3*_ *5.1*, *qSER5.1,* and *qFLL5.1*: this study under SF and control
*qDTF6.1*	*QHd6b*: (Li et al. 2003); (Mei et al. 2003); *qDTF6.1*: this study under control; *qFLL6.1*: this study under SF
*qDTF10.1*	(Price et al. 1997); (Li et al. 1998); *qDTF10.1*:this study under control

**Table 6:** Genomic positions of QTLs identified in control irrigated condition with its QTL cluster and comparison to the QTLs identified in stagnant flooding in this study and previously published QTLs

QTL	Studies shared common region
*qGY12.1*	*qtl12.1*: (Bernier et al. 2007)
*qGW1.1*	*GW1-2*: (Yu et al. 2008); *qGW1.1*: this study under SF
*qGW2.1*	*gw-2*: (Lu et al. 1997); *QKw2b*: (Li et al. 1997); *qGW-2*: (Cho et al. 2003); *Kw2-2*: (Gao et al. 2004); *qLSL*_*3*_ *2.1*: this study under control
*qGW2.2*	*QKw2a*: (Li et al. 1997); *qTGWT-2-2*: (Zhuang et al. 2002); *tgw2*: (Ishimaru 2003); *gw2.1*: (Thomson et al. 2003); *qTGW-2a*: (Zou et al. 2005)
*qGW5.1*	*gw-5*: (Lu et al. 1997); *QKw5*: (Li et al. 1997); *qGW5.1*: this study under SF
*qHI3.1*	*qhi3.3/qhi3.4/qhi3.5*: (Lanceras et al. 2004); *qFLW3.1, qDTH3.1*: this study under control and SF; *qLSL*_*1*_ *3.1*, *qBM3.1*: this study under control
*qHI7.1*	none
*qTN3.1*	*TN(R3)*: (Kobayashi et al. 2003); *qPN3.1*, *qLSL*_*3*_ *3.1*: this study under control
*qPN1.1*	*PnN(R3-1)*: (Kobayashi et al. 2003); *npp(1.1)*: Nagata et al. 2002; *qBM1.1*: this study under control
*qPN3.1*	*PN(3)*: (Jiang et al. 2004); *qFLW3.1*, *qDTH3.1*: this study under control and SF; *qTN3.1*, *qLSL*_*3*_ *3.1*: this study under control
*qBM1.1*	*PW(1)*: (Lian et al. 2005); *qPN1.1*: this study under control
*qBM3.1*	*qHI3.1*; *qLSL*_*1*_ *3.1*: this study under control
*qPL1.1*	(Mei et al. 2005)
*qPL2.1*	*pl2*: (Xing et al. 2001); *qPL2-1*: (Cui et al. 2002); (Mei et al. 2003) 3; *qFLL2.1*: this study under control
*qPL6.1*	(Mei et al. 2003); *PL(6)*: (Jiang et al. 2004) J; *qSER6.1*, *qLSL*_*3*_ *6.1*: this study under control
*qPL7.1*	(Mei et al. 2005)
*qPL9.1*	*pl9*: (Xing et al. 2001); *qPL-9*: (Cho et al. 2003); *pl9.1*: (Septiningsih et al. 2003); *pl9.1*: (Thomson et al. 2003); (Marri et al. 2005); (Li et al. 2006); *qPL9.1*: this study under SF
*qPH1.1*	(Li et al. 1998); (Mei et al. 2003); *qLSL*_*1*_ *1.1*: this study under control
*qPH3.1*	(Li et al. 1997); *QPh3a*: (Li et al. 2003)
*qPH5.1*	(Mei et al. 2003); *ph5.1*: (Moncada et al. 2001); (Thomson et al. 2003); *qPH5.1*: this study under SF; *qLSL*_*1−*_ *5.1, qLSL*_*2*_ *5.1, qLSL*_*3*_ *5.1, qSER5.1, qFLL5.1*: this study under control and SF
*qSER5.1*	*qSER5.1*: this study under SF; *qPH5.1*, *qLSL*_*1−*_ *5.1, qLSI2-5.1*, *qLSL*_*2*_ *5.1*, *qFLL5.1*: this study under control and SF
*qSER6.1*	*qSER6.1*: this study under SF; *qPL6.1, qLSL*_*3*_ *6.1*: this study under control
*qLSL*_*1*_ *1.1*	*qPH1.1*: this study under control
*qLSL*_*1*_ *3.1*	*qBM3.1*, *qHI3.1*: this study under control
*qLSL*_*1*_ *5.1*	*qLSL*_*1*_ *5.1*: this study under SF; *qPH5.1, qLSL*_*2*_ *5.1*, *qLSL*_*3*_ *5.1, qSER5.1*, *qFLL5.1*: this study under control and SF
*qLSL*_*2*_ *3.1*	none
*qLSL*_*2*_ *5.1*	*qLSL*_*2*_ *5.1*: this study under SF; *qPH5.1, qLSL*_*1*_ *5.1*, *qLSL*_*3*_ *5.1, qSER5.1*, *qFLL5.1*: this study under control and SF
*qLSI*_*2*_ *9.1*	none
*qLSL*_*3*_ *2.1*	*qGW2.1*: this study under control
*qLSL*_*3*_ *3.1*	*qTN3.1*, *qPN3.1*: this study under control
*qLSL*_*3*_ *5.1*	*qLSL*_*3*_ *5.1*: this study under SF; *qPH5.1, qSER5.1, qLSL*_*1*_ *5.1, qLSL*_*2*_ *5.1, qFLL5.1*: this study under control and SF
*qLSL*_*3*_ *6.1*	*qSER6.1*: this study under control and SF; *qPL6.1*: this study under control
*qLSI*_*3*_ *8.1*	none
*qLSL*_*3*_ *11.1*	none
*qFLL1.1*	(Mei et al. 2005)
*qFLL2.1*	*qPL2.1*: this study under irrigated
*qFLL4.1*	(Mei et al. 2005)
*qFLL5.1*	*QFll7*: Yue et al. 2006; Yan et al. 2003; *qFLL5.1*: this study under SF; *qPH5.1*, *qLSL*_*1*_ *5.1, qLSL*_*2*_ *5.1, qLSL*_*3*_ *5.1*, *qSER5.1*: this study under irrigated and SF
*qFLW3.1*	(Mei et al. 2005); *qFLW3.1*: this study under SF; *qHI3.1, qBM3.1*, and *qLSL*_*1*_ *3.1*: this study under irrigated); *qDTF3.1*: this study under irrigated and SF
*qFLW4.1*	*QFlw4*: (Bing et al. 2006); *flw4*: (Yan et al. 1999)
*qFLW5.1*	none
*qFLW12.1*	none
*qFLW12.2*	(Mei et al. 2005)
*qDTF3.1*	(Albar et al. 1998); *dth3.1*: (Moncada et al. 2001); *qHD-3-1*: (Takeuchi et al. 2001); *Hd9*: (Lin et al. 2002) 2; *qHDD3-1*: (Hittalmani et al. 2003); *QHd3a*: (Li et al. 2003); (Mei et al. 2003); *qEMF3*: (Hirabayashi et al. 2014); *qDTF3.1*: this study under SF; *qFLW3.1*: this study under control and SF; *qHI3.1, qBM3.1*, and *qLSL*_*1*_ *3.1*: this study under control
*qDTF6.1*	*QHd6b*: (Li et al. 2003); (Mei et al. 2003); *qDTF6.1*: this study under SF
*qDTF10.1*	(Price et al. 1997); Li et al. (1998)); *qDTF10.1*: this study under SF

#### QTLs for GY, GW, HI, and SR

Three QTLs were detected for grain yield under stagnant flooding, *qGY3.1*, *qGY5.1* and *qGY6.1.* (Table 3, Fig. 1). It is noteworthy that the largest QTL, *qGY3.1* with an LOD score of 6.2 and phenotypic variation (R^2^) of 14.3% was detected on the top of chromosome 3S as a cluster with four other QTLs: *qDTF3.1*, which was detected in both environments, *qGW3.1*, *qFLW3.1*, and *qPH3.1*. Interestingly, the alleles that increase the effects of all the QTLs in this cluster came from Ciherang-Sub1. *qGY3.1* was also detected in similar regions as previous reported by Xiao et al. (1996)) under irrigated condition, (Moncada et al. 2001) under upland cultivation, and (Bernier et al. 2007) under drought condition (Table 5). Another QTL, *qGY5.1* (LOD = 3.66, R^2^ = 12.2%) was in a cluster with two other QTLs of different traits on the upper part of chromosome 5 L, *qGW5.1*, which was detected in both environments, and *qHI5.1*. However, the increased-alleles of *qGY5.1* and *qHI5.1* were from IR10F365, while that of *qGW5.1* was from Ciherang-Sub1 (Table 3, Fig. 1). *qGY5.1* was also detected in the similar region as *qYl-5* (Cho et al. 2003). Under control conditions, only one QTL for grain yield, *qGY12.1* was detected (LOD = 3.39 and R^2^ = 9.3) (Table 4, Fig. 2); this QTL partially overlapped with *qFLW12.1* and in the same region as a major QTL for yield under drought (Bernier et al. 2007) (Table 6). It is interesting to note that some of the GY QTLs detected in this study were in similar positions as previously identified GY QTLs, under different environments. However, further research is needed to determine whether they are governed by the same gene(s) or they are simply closely linked.

Six QTLs were detected for 100-grain weight under SF on chromosomes 1, 2, 3, 5, and 10 (2 QTLs). All alleles with increasing effects were from Ciherang-Sub1 except for the largest QTL, *qGW10.2* (LOD = 6.30 and R^2^ = 13.1%) on chromosome 10S (Table 3, Fig. 1). *qGW1.1* was also detected as the largest QTL for GW under control conditions in this study (LOD = 5.34 and R^2^ = 15.2%) and co-localized with *GW1-1* (Yu et al. 2008) (Table 5). *qGW2.1* was in the similar region as *tgw2* (Yoon et al. 2006) and in a cluster with *qLSI3-2.1* and *qHI2*; however, the increased-allele for *qHI2* was from IR10F365. *qGW3.1* was co-localized with *QKw3a* (Li et al. 1997) and in a cluster with other four QTLs detected on chromosome 3S. *qGW5.1* was in the similar region as *gw-5* (Lu et al., 1997); the same QTL was also detected in the same position in the control and in a cluster with other two QTLs on the upper part of chromosome 5 L. *qGW10.1* was co-localized with *QKw10* (Li et al. 1997); while *qGW10.2* was in the similar regions of the QTLs detected by Ishimaru (2003) and Thomson et al. (2003). Under control conditions, four QTLs were detected on chromosomes 1, 2 (2 QTLs) and 5. In all cases the source of the increased alleles were from Ciherang-Sub1 (Table 4, Fig. 2). The two QTLs on chromosome 2 were co-localized with many previous reported QTLs; in addition, *qGW2.1* was in a similar region of *qLSI*_*3*_ *2.1* (Table 6).

Four harvest index QTLs were detected under stagnant flooding located on chromosomes 2, 5 and 7 (2 QTLs). The largest QTL, *qHI7.2* (LOD = 8.67 and R^2^ = 20.4%) was the only one that had the increased allele from IR10F365 (Table 3, Fig. 1). This QTL was co-localized with two other QTLs, *qPL7.1* and *qFLW7.1*; however, the increased-allele of *qFLW7.1* was from Ciherang-Sub1. Under control conditions, two QTLs were identified on chromosomes 3 and 7, with the largest one, *qHI3.1*, detected with a LOD of 4.91 and R^2^ of 14.2% (Table 4, Fig. 2). On the other hand, only two minor QTLs for survival rate under stagnant flooding were identified on chromosomes 1 and 6, *qSR1.1* (LOD = 2.89 and R^2^ = 10.0%) with the beneficial allele from IR10F365 and *qSR6.1* (LOD = 2.97 and R^2^ = 7.3%) with that of Ciherang-Sub1, respectively (Table 3, Fig. 1). *qSR6.1* was in a cluster with three other QTLs, *qSER6.1*, which was detected in both environments, *qPH6.1*, and *qLSL*_*2*_ *6.1*; however, the increased-allele in *qPH6.1* was from IR10F365.

#### QTLs for DTF, PH, SER and PL

Five QTLs were detected for days to flowering under SF on chromosomes 1, 3, 5, 6, and 10 (Table 3, Fig. 1). Interestingly, the three QTLs on chromosomes 3, 6 and 10 were also mapped in similar regions under control conditions (Table 4, Fig. 2). The largest QTL was *qDTF3.1* with an increased allele or lateness from Ciherang-Sub1 (LOD = 12.51 and R^2^ = 26.0% in stress; LOD = 21.81 and R^2^ = 42.19% in the control); while the other two QTLs, *qDTF6.1* and *qDTF10.1*, with lateness from IR10F365. *qDTF5.1* was strongly detected by both software but only under SF, on the lower part of chromosomes 5L (LOD = 4.11 and R^2^ = 7.0%) in a cluster with six other QTLs, *qLSL*_*1*_ *5.1*, *qLSL*_*2*_ *5.1*, *qLSL*_*3*_ *5.1*, *qSER5.1*, *qPH5.1*, and *qFLL5.1* (Table 5). All of the QTLs for DTF detected in this study were previously reported in similar positions by other researchers (Tables 5 and 6).

Three QTLs were detected for plant height on chromosomes 3, 5 and 6 under SF, with the largest, *qPH5.1* (LOD = 5.59 and R^2^ = 12.1%) on the lower arm of chromosome 5 L with an increased-allele from IR10F365 (Table 3, Fig. 1). Under control conditions, the same QTL was detected with larger effect (LOD = 9.90 and R^2^ = 23.9%) (Table 4, Fig. 2). The QTL on chromosome 3, *qPH3.1*, was also detected under control condition with partly overlapping position; with an increased effect from Ciherang-Sub1. A minor QTL, *qPH1.1* was also detected on chromosome 1 under control conditions, with an increased allele effect from Ciherang-Sub1. All of the PH QTLs detected in this study were previously reported in similar positions by other researchers (Tables 5 and 6). On a related trait, two QTLs were detected for shoot elongation rate under SF on chromosomes 5 and 6. The largest QTL, *qSER5.1* (LOD = 12.46 and R^2^ = 32%) positioned in a cluster of QTLs on the lower part of chromosome 5L (Table 3, Fig. 1), with an increased allele from IR10F365. The second QTL, *qSER6.1* was a minor one with the increased-allele effect from Ciherang-Sub1. It was worth noting that under control, both QTLs were mapped at the same positions on chromosomes 5 and 6 (Table 4, Fig. 2).

Under SF, two QTLs for panicle length *qPL7.1* and *qPL9.1* were identified with increase alleles from IR10F365 (Table 3, Fig. 1). *qPL7.1* was the largest with an LOD value of 5.47 and R^2^ of 22%. Under control condition, five QTLs were detected on chromosomes 1, 2, 6, 7 and 9 (Table 4, Fig. 2). Three of them had increased alleles from IR10F365, including the largest QTL *qPL2.1* (LOD = 7.11 and R^2^ = 14.31%). *qPL9.1* was detected under both SF and control, sharing the same region on the lower arm of chromosome 9 L. The panicle length QTLs detected in this study have also been previously mapped in similar positions (Tables 5 and 6).

#### QTLs for FLL and FLW

Four QTLs were identified for flag leaf length under SF on chromosomes 4, 5, 6, and 9 (Table 3, Fig. 1). Three of the QTLs had increased alleles from IR10F365, including the largest QTL *qFLL5.1* (LOD = 6.18; R^2^ = 14.0%) positioned in a cluster with other QTLs on the lower part of chromosome 5L; the same QTL was also detected under control conditions. Under the control, four QTLs were identified on chromosomes 1, 2, 4, and 5, with three of them had increased alleles from IR10F365 (Table 4, Fig. 2). For flag leaf width, two QTLs, *qFLW3.1* (LOD = 4.00 and R^2^ = 11.3%) and *qFLW7.1* (LOD = 3.11 and R^2^ = 14.0%) were detected under SF with their increased allele from Ciherang-Sub1 (Table 3, Fig. 1). Under the control, 5 QTLs were detected on chromosomes 3, 4, 5 and 12 (2 QTLs), *qFLW12.1* being the only QTL having increased allele from IR10F365 (Table 4, Fig. 2). *qFLW4.1* was the largest under the control, with an LOD score of 8.88 and R^2^ of 20.11%. The positions of *qFLW3.1* mostly overlap under SF and control conditions. Most of the FLL and FLW QTLs were previously mapped in similar regions (Tables 5 and 6).

#### QTLs for LSL

Under SF, only one QTL for leaf sheath length for the first internode, *qLSL*_*1*_ *5.1* with increased allele from IR10F365, was detected (LOD = 4.44 and R^2^ = 11.0%) in a cluster of QTLs, including *qLSL*_*2*_ *5.1 and qLSL*_*3*_ *5.1*, on the lower arm of chromosome 5 L (Table 3, Fig. 1). Three QTLs were identified under control on chromosomes 1, 3, and 5 (Table 4, Fig. 2). *qLSL*_*1*_ *5.1* was detected in the same position as in SF condition as the largest QTL in irrigated (LOD = 5.33 and R^2^ = 13.1%). The other two QTLs had increased alleles from Ciherang-Sub1. Meanwhile for LSL_2_, two QTLs were identified on chromosomes 5 and 6 under SF, with the increased alleles from IR10F365 (Table 3, Fig. 1). *qLSL*_*2*_ *5.1* was the largest QTL (LOD = 4.96 and R^2^ = 14.3%). Under control, three QTLs were detected on chromosomes 3, 5, and 9 (Table 4, Fig. 2). Two of the QTLs had increased alleles effects from IR10F365, including *qLSL*_*2*_ *5.1*, which was also identified at the same position as under SF. This QTL was also the largest QTL under control (LOD = 4.95 and R^2^ = 11.2%). Two QTLs for LSL_3_ were detected under SF, located on chromosomes 2 and 5 with the increased allele from Ciherang-Sub1 and IR10F365, respectively (Table 3, Fig. 1). The largest QTL was *qLSL*_*3*_ *2.1* on chromosome 2 (LOD = 4.16 and R^2^ = 10.0%). *qLSL*_*3*_ *5.1* was mapped on a cluster of QTLs on the lower part of chromosome 5L. Meanwhile, under control, six QTLs were identified and four of the QTLs had increased alleles from Ciherang-Sub1 (Table 4, Fig. 2). The LOD values of the QTLs ranged from 4.40 – 7.41 and R^2^ of 9.26 – 14.72%. The largest QTL was *qLSL*_*3*_ *8.1*, with the increased allele from IR10F365. *qLSL*_*3*_ *2.1* and *qLSL*_*3*_ *5.1* were also mapped in similar positions on chromosomes 2 and 5, respectively, as in SF environment. Some of these LSL traits were mapped in a cluster with other traits (Tables 5 and 6).

#### QTLs for TN, PN, and BM

There were no QTLs detected for tiller number, panicle number and shoot biomass under stagnant flooding. While under control conditions, only one QTL detected for tiller number on top of chromosome 3S, *qTN3.1* (LOD = 4.29, R^2^ = 12.5%), with increased allele from IR10F365, in a cluster with some other QTLs, *qPH3.1*, *qHI3.1, qPN3.1, qBM3.1,* and *qLSL*_*3*_ *3.1* (Table 4, Fig. 2). Two QTLs were identified for panicle number under control on chromosomes 1 and 3 with the increased alleles from IR10F365. *qPN1.1* (LOD = 3.49 and R^2^ = 10.3%) was detected in a cluster with some other QTLs *qPH1.1*, *qGW1.1*, *qBM1.1*, and *qLSL*_*1*_ *1.1*, on the lower part of chromosome 1S. Two QTLs were detected under control for shoot biomass, *qBM1.1* (LOD = 3.92 and R^2^ = 10.2%) and *qBM3.1* (LOD = 3.33 and R^2^ = 9.9%), with the increased-alleles from IR10F365 and Ciherang-Sub1, respectively. Similar QTLs for the three traits had been previously detected in the similar regions (Tables 5 and 6).

Our results demonstrated that combinations of three or two yield QTLs gave better performances under stagnant flooding conditions (Table 7). Under control conditions, however, *qGY12* only had a small contribution for increasing yield (7.2%; Table 8). A group of RILs having combinations of three QTLs gave the best performance under stagnant flooding with an increased yield of 89.2, 56.1, 44.3, 51.5, 30.6, 17.2, and 37.6% compared to the RIL that did not possess any of the yield QTLs, only *qGY6* from Ciherang-Sub1, only *qGY5* from IR10F365, only *qGY3* from Ciherang-Sub1 (detected in a cluster with four different QTLs referred as C3S QTLs in Table 7), *qGY5* and *qGY6*, *qGY3* and *qGY6*, and *qGY3* and *qGY5*, respectively. In general the groups having higher yield under SF also are taller, have more tillers and panicles, longer flag leaf, more biomass, higher shoot elongation rate, longer leaf sheaths, and higher survival rate. A similar cluster of QTLs on the top of chromosome 3 (C3S QTLs; Table 8), however, hardy had any effect in yield under control; either alleles contributed similarly to yield. This might be due to the fact that the beneficial alleles of those QTLs detected in the control were mix from both parents (Tables 7 and 8; Fig. 2). On the other hand, a cluster of seven different QTLs on the lower part of chromosome 5L just below *qGY5* (C5L QTLs; Table 7) revealed that these QTLs indirectly contribute to a yield increase of 19.5% under stagnant flooding; which is partly due to increases in plant height, elongation rate, leaf sheath length, flag leaf length, and earliness from IR10F365 alleles. A similar cluster of six QTLs was also detected in the same region under control; in which this genomic region of IR10F365 indirectly increased yield by 6.1% (C5L QTLs; Table 8). Additionally, a smaller cluster of QTLs close to the centromeric region of chromosome 6 with increased alleles from Ciherang-Sub1 (C6C QTLs; Tables 7 and 8) contributed to a yield increase of 11.9 and 5.1% under SF and control, respectively.

**Table 7:** Phenotypic effect of grain yield QTLs and QTL clusters identified in stagnant flooding condition

*qGY3* and C3S QTLs^a)^	*qGY5*	*qGY6*	C5L QTLs^c)^	C6C QTLs^d)^	#RIL	DTF (d)	PH (cm)	TN	PN	FLL (cm)	FLW (cm)	PL (cm)	BM (g/m^2^)	SER (cm/d)	HI	GW (g)	LSL_1_ (cm)	LSL_2_ (cm)	LSL_3_ (cm)	GY (kg/ha)	SR (%)
+	+	+	NA	NA	28	96	134.2	7	7	29.8	1.83	25.8	1037.1	1.35	0.35	2.67	14.9	18.0	28.6	3408	71.2
+	+	-	NA	NA	12	97	129.8	7	6	28.7	1.77	26.1	907.4	1.32	0.30	2.66	14.6	17.8	28.6	2476	67.0
+	-	+	NA	NA	25	97	134.9	7	6	29.3	1.83	26.3	1070.3	1.39	0.28	2.76	14.6	18.0	28.2	2908	71.7
-	+	+	NA	NA	35	93	127.7	7	6	30.5	1.76	25.1	984.8	1.42	0.28	2.60	14.2	17.5	28.4	2610	68.6
+	-	-	NA	NA	8	97	128.2	6	5	27.5	1.86	25.6	914.2	1.31	0.25	2.79	13.1	16.7	29.5	2249	71.3
-	+	-	NA	NA	8	97	122.6	6	6	28.4	1.74	25.8	888.8	1.23	0.29	2.56	14.3	16.3	25.2	2361	66.9
-	-	+	NA	NA	19	95	126.6	7	6	29.8	1.79	25.0	967.7	1.30	0.26	2.68	14.0	17.8	27.9	2183	64.0
-	-	-	NA	NA	1	97	119.2	4	4	27.4	1.84	27.3	544.7	1.03	0.28	3.14	12.5	14.3	21.0	1801	58.3
NA^b)^	NA	NA	+	NA	83	95	132.7	7	6	30.2	1.80	25.7	1013.6	1.43	0.30	2.65	14.8	18.2	28.7	2856	69.3
NA	NA	NA	-	NA	32	97	125.2	7	6	28.1	1.78	25.5	951.6	1.21	0.26	2.72	13.1	16.4	26.0	2390	67.3
NA	NA	NA	NA	+	74	95	130.2	7	7	30.2	1.81	26.0	1001.6	1.39	0.30	2.68	14.4	17.6	28.3	2879	69.7
NA	NA	NA	NA	-	32	97	131.1	7	6	28.7	1.77	25.6	997.1	1.30	0.28	2.64	14.7	17.6	28.0	2572	67.7

^a)^a cluster of QTLs on the top of chromosome 3S

^b)^not applied

^c)^a cluster of QTLs on the lower part of chromosome 5L just below *qGY5*

^d)^a cluster of QTLs close to the centromeric region of chromosome 6

**Table 8:** Phenotypic effect of grain yield QTL and QTL clusters identified in irrigated control conditions

qGY12	C3S QTLs^b)^	C5L QTLs^c)^	C6C QTLs^d)^	#RIL	DTF	PH	TN	PN	FLL	FLW	PL	BM	PER	HI	GW	LSL_1_	LSL_2_	LSL_3_	GY
+	NA	NA	NA	32	90	124.0	11	11	31.5	1.88	25.8	1372.2	1.33	0.44	2.71	13.9	21.6	31.5	5910
-	NA	NA	NA	80	89	126.3	10	10	32.2	1.93	26.2	1323.3	1.35	0.43	2.74	14.1	22.6	33.2	5511
NA^a)^	+	NA	NA	77	91	126.7	10	10	32.5	1.89	25.9	1358.8	1.31	0.42	2.73	14.9	21.9	33.2	5588
NA	-	NA	NA	67	87	124.2	10	10	31.4	1.97	26.4	1290.1	1.37	0.45	2.73	13.4	22.6	32.2	5671
NA	NA	+	NA	83	88	128.3	10	10	32.6	1.96	26.4	1321.8	1.40	0.44	2.72	14.9	22.6	33.4	5729
NA	NA	-	NA	32	90	120.4	10	10	30.8	1.85	25.5	1359.0	1.22	0.42	2.77	13.2	20.8	31.3	5399
NA	NA	NA	+	74	88	125.9	10	10	32.8	1.95	26.6	1337.2	1.37	0.44	2.75	14.2	22.3	32.8	5749
NA	NA	NA	-	32	91	126.2	10	10	31.1	1.85	25.5	1350.7	1.26	0.42	2.70	14.7	22.1	32.0	5470

^a)^not applied

^b)^a cluster of QTLs on the top of chromosome 3S

^c)^a cluster of QTLs on the lower part of chromosome 5L just below *qGY5*

^d)^a cluster of QTLs close to the centromeric region of chromosome 6

## Conclusions

This study reports the first effort for mapping beneficial QTLs under stagnant flooding conditions. In contrast to most Sub1-lines developed to date, Ciherang-Sub1 was found to be tolerant to stagnant flooding and many beneficial QTLs were derived from this variety. Future research in stagnant flooding tolerance should target these valuable QTLs after validation of trait data by conducting additional field trials across years and locations, especially on the distal part of chromosome 3 (*qGY3* and 4 other QTLs), *qGY5*, *qGY6*, clusters of QTLs on the lower part of chromosome 5L and around the centromeric region of chromosome 6. These QTLs can be further investigated for molecular studies and for use in molecular breeding. In addition, since the two parents are elite breeding material, the best yielding lines that performed well in each environment can be further evaluated through multi-environmental trials. It should be noted that all of these RILs are already fixed for the *SUB1* gene, which provides additional protection from complete submergence in both stagnant flooding and irrigated environments.

## Additional files

Additional file 1: Table S1. List of traits measured in both stagnant flooding and irrigated control conditions. (DOCX 13 kb)

Additional file 2: Table S2. Performances of selected RILs in stagnant flooding and irrigated control IRRI fields in 2014 WS. (DOCX 13 kb)

Additional file 3: Figure S1. Percentage difference between means of trait values of 25 highest and lowest yielding lines relative to the mean of the population under stagnant flooding condition. (PDF 87 kb)

Additional file 4: Figure S2. Percentage difference between means of trait values of 25 highest and lowest yielding lines relative to the mean of the population under irrigated control condition. (PDF 85 kb)

Additional file 5: Figure S3. Relationship between shoot elongation rate (SER) under stagnant flooding condition, relative grain yield (ratio of yield under SF to that in the control) and survival rate (SR). (PPTX 46 kb)

Additional file 6: Table S3. Correlations among traits under stagnant flooding condition. (DOCX 15 kb)

Additional file 7: Table S4. Correlations among traits under irrigated control condition. (DOCX 15 kb)
