# Supplementary material for: Identification of QTLs for yield and agronomic traits in rice under stagnant flooding conditions
Source: Rice (N Y). 2017 Apr 20;10:15. doi: 10.1186/s12284-017-0154-5 (PMC5398972; doi:10.1186/s12284-017-0154-5)
Supplement: Supplementary file 1 — List of traits measured in both stagnant flooding and irrigated control conditions. (DOCX 13 kb) [file 12284_2017_154_MOESM1_ESM.docx]

Supplementary Table 1: List of traits measured in both stagnant flooding and irrigated control conditions.

| **Traits** | **Description** |
| --- | --- |
| *Days to flowering* (DTF in days) | recorded when 50% of plants in each plot exerted their panicles |
| *Plant height* (PH in cm) | measured starting from the base of the plant to the tip of the panicle during maturity stage by sampling 5 plants randomly from the second row of each plot |
| *Shoot elongation rate* (SER in cm/day) | evaluated by measuring the height of the same 5 plants per plot at five different dates from the base of the plant to the tip of the panicle, starting from 35 day after transplanting (DAT) to 63 DAT, by keeping 7 days interval between each set of reading; then taken average from the cumulative readings |
| *Number of tillers* (TN) | counted from the same 5 plants per plot at heading stage |
| *Number of panicles* (PN) | counted for the same 5 plants per plot |
| *100 grain weight* (GW in g) | measured by weighing100 grains per 5 plants per plot after threshing and oven drying the panicles |
| *Shoot biomass* (BM in g/m^2^) | measured at maturity stage by harvesting above-ground biomass of 5 plants near to the edge at the ground level from each plot; straws and panicles separated and oven-dried at 50° C for three days, weighed, and added to calculate shoot biomass |
| *Flag leaf length* (FLL in cm) | measured from the base to the tip of the leaf blade for 3 of the 5 plants selected |
| *Flag leaf width* (FLW in cm) | measured of the same flag leaf along the maximum width of the leaf blade for the same 3 plants |
| *Panicle length* (PL in cm) | measured for the same 3 plants at flowering stage |
| *Harvest index* (HI) | calculated as grain yield/total aboveground biomass; plants harvested at maturity stage |
| *Leaf sheath length for the 1^st^ internode* (LSL_1_ in cm) | measured an individual rice tiller of the 3 plants per plot at the flowering stage |
| *Leaf sheath length for the 2^nd^ internode* (LSL_2_ in cm) | measured an individual rice tiller of the 3 plants per plot at the flowering stage |
| *Leaf sheath length for the 3^rd^ internode* (LSL_3_ in cm) | measured an individual rice tiller of the 3 plants per plot at the flowering stage |
| *Grain yield per plot* (GY in kg ha^-1^) | measured by threshing and oven-drying the bulk-harvested grains per plot for both replications for three days at 50^o^C to reduce the moisture content (MC) which measured using a grain moisture meter; final yield measured by taking into account the MC values |
| *Survival rate* (SR) | recorded as % of total no. of plants survived per plot after growing in stagnant water condition |
