# Supplementary material for: Identification of QTLs for yield and agronomic traits in rice under stagnant flooding conditions
Source: Rice (N Y). 2017 Apr 20;10:15. doi: 10.1186/s12284-017-0154-5 (PMC5398972; doi:10.1186/s12284-017-0154-5)
Supplement: Supplementary file 2 — Performances of selected RILs in stagnant flooding and irrigated control IRRI fields in 2014 WS. (DOCX 13 kb) [file 12284_2017_154_MOESM2_ESM.docx]

Supplementary Table 2: Performances of selected RILs in stagnant flooding and irrigated control IRRI fields in 2014 WS.

| **RIL IDs** | **DTF (d)** | **PH (cm)** | **TN** | **PN** | **FLL (cm)** | **FLW (cm)** | **PL (cm)** | **BM (g/m^2^)** | **SER (cm/d)** | **HI** | **GW (g)** | **LSL_1_ (cm)** | **LSL_2_ (cm)** | **LSL_3_ (cm)** | **GY (kg/ha)** | **SR (%)** | **Note** |
| --- | --- | --- | --- | --- | --- | --- | --- | --- | --- | --- | --- | --- | --- | --- | --- | --- | --- |
| 214 | 102 | 138.5 | 7 | 7 | 28.4 | 1.95 | 25.8 | 1281.0 | 1.31 | 0.46 | 2.59 | 12.8 | 14.3 | 26.5 | 5184 | 60.2 | best-SF stress |
| 186 | 88 | 124.3 | 9 | 8 | 31.2 | 2.17 | 24.0 | 1232.2 | 1.30 | 0.60 | 2.76 | 14.0 | 22.9 | 29.3 | 7398 | NA | best- SF control |
| 182 | 93 | 140.1 | 6 | 6 | 35.4 | 1.86 | 26.6 | 1199.7 | 1.59 | 0.37 | 2.43 | 15.9 | 19.2 | 34.4 | 4067 | 75.2 | 6^th^ best-stress |
| 182 | 86 | 134.9 | 9 | 9 | 32.4 | 2.09 | 27.5 | 1391.3 | 1.42 | 0.48 | 2.57 | 13.3 | 25.6 | 40.5 | 6681 | NA | 6^th^ best-control |
| 189 | 102 | 111.7 | 7 | 7 | 26.1 | 1.51 | 23.4 | 574.3 | 0.89 | 0.14 | 2.68 | 9.2 | 13.7 | 23.7 | 921 | 44.9 | worst-SF stress |
| 299 | 90 | 135.4 | 11 | 10 | 32.1 | 2.09 | 25.5 | 1672.2 | 1.40 | 0.22 | 2.77 | 16.7 | 22.2 | 36.5 | 3600 | NA | worst-SF control |
