# Supplementary material for: Identification of QTLs for yield and agronomic traits in rice under stagnant flooding conditions
Source: Rice (N Y). 2017 Apr 20;10:15. doi: 10.1186/s12284-017-0154-5 (PMC5398972; doi:10.1186/s12284-017-0154-5)
Supplement: Supplementary file 3 — Percentage difference between means of trait values of 25 highest and lowest yielding lines relative to the mean of the population under stagnant flooding condition. (PDF 87 kb) [file 12284_2017_154_MOESM3_ESM.pdf]

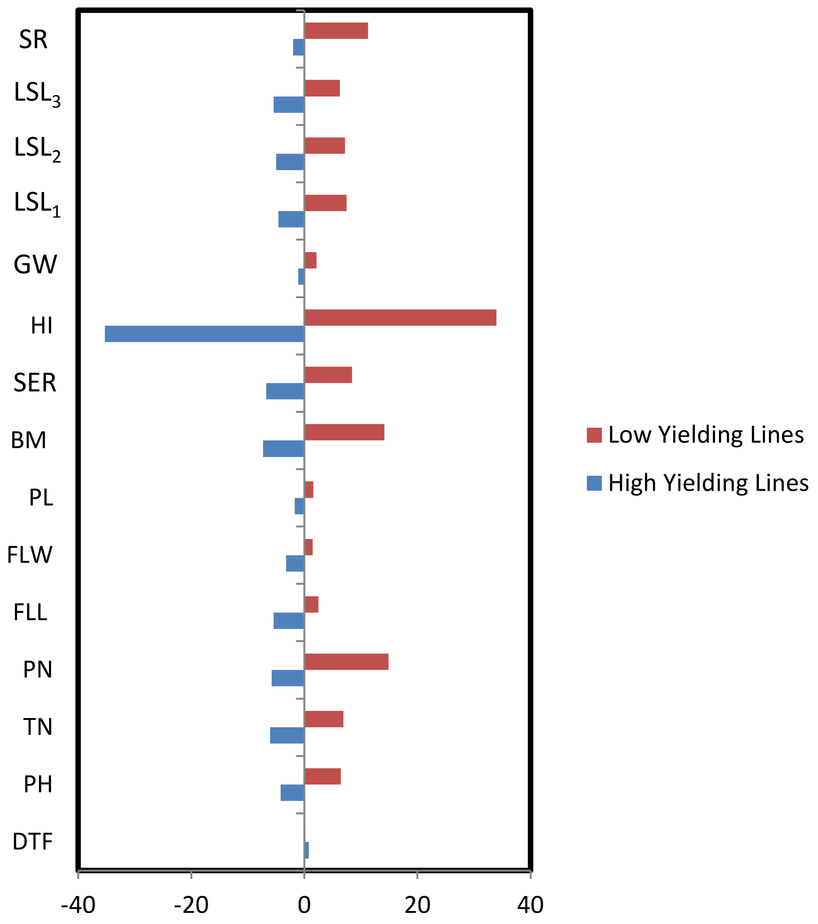

Additional file 3: Figure S1. Percentage difference between means of trait values of 25 highest and lowest yielding lines relative to the mean of the population under stagnant flooding condition
