# Supplementary material for: Identification of QTLs for yield and agronomic traits in rice under stagnant flooding conditions
Source: Rice (N Y). 2017 Apr 20;10:15. doi: 10.1186/s12284-017-0154-5 (PMC5398972; doi:10.1186/s12284-017-0154-5)
Supplement: Supplementary file 5 — Relationship between shoot elongation rate (SER) under stagnant flooding condition, relative grain yield (ratio of yield under SF to that in the control) and survival rate (SR). (PPTX 46 kb) [file 12284_2017_154_MOESM5_ESM.pptx]

## Slide 1
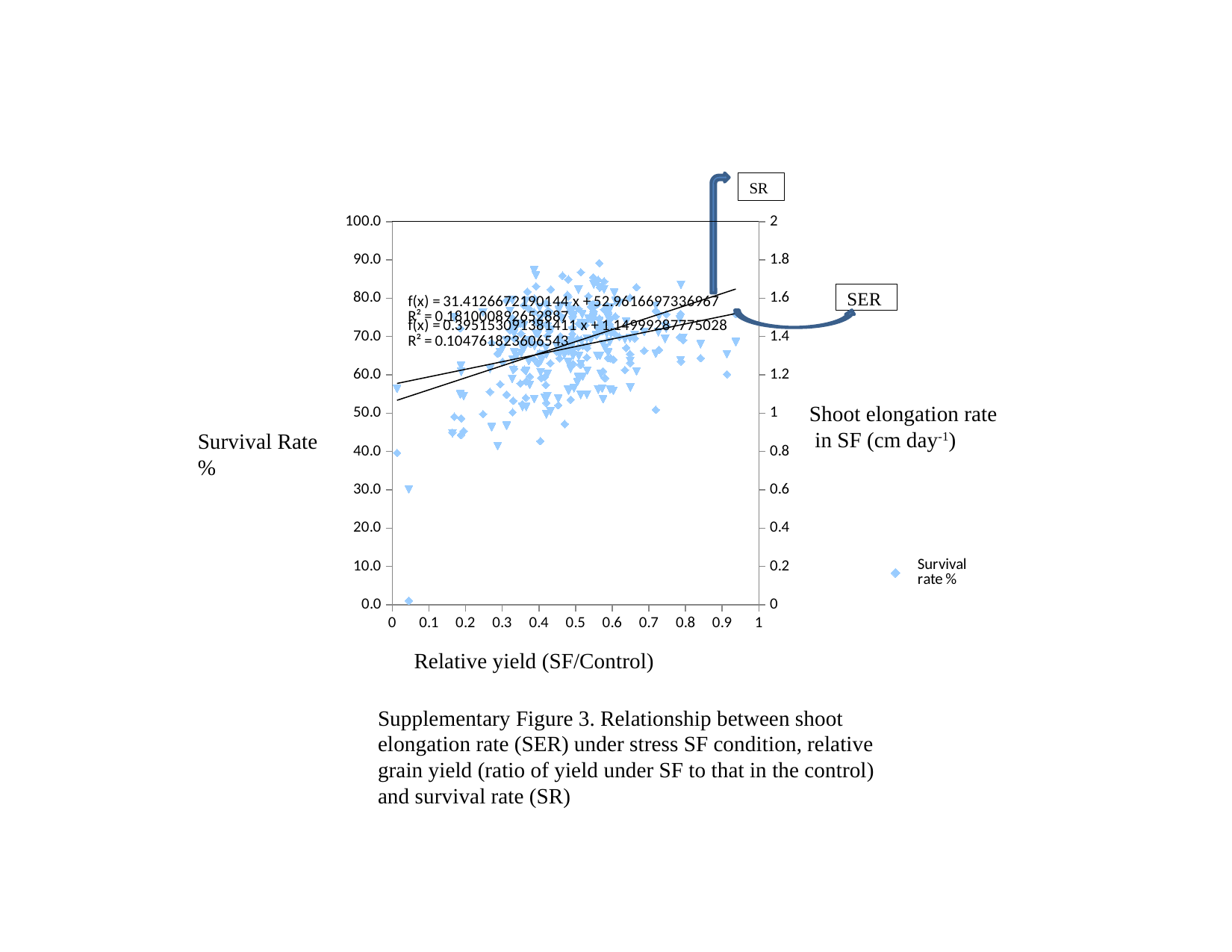

SR
### Chart
| Category | Survival rate % | PER Stress |
|---|---|---|Shoot elongation rate
 in SF (cm day-1)
Survival Rate %
Relative yield (SF/Control)
Supplementary Figure 3. Relationship between shoot elongation rate (SER) under stress SF condition, relative grain yield (ratio of yield under SF to that in the control) and survival rate (SR)
