# Supplementary material for: Identification of QTLs for yield and agronomic traits in rice under stagnant flooding conditions
Source: Rice (N Y). 2017 Apr 20;10:15. doi: 10.1186/s12284-017-0154-5 (PMC5398972; doi:10.1186/s12284-017-0154-5)
Supplement: Supplementary file 6 — Correlations among traits under stagnant flooding condition. (DOCX 15 kb) [file 12284_2017_154_MOESM6_ESM.docx]

Supplementary Table 3: Correlations among traits under stagnant flooding condition.

| **Trait** | **DTF** | **PH** | **TN** | **PN** | **FLL** | **FLW** | **PL** | **BM** | **SER** | **HI** | **GW** | **LSL_1_** | **LSL_2_** | **LSL_3_** | **GY** | **SR** |
| --- | --- | --- | --- | --- | --- | --- | --- | --- | --- | --- | --- | --- | --- | --- | --- | --- |
| **DTF** | 1.000 |  |  |  |  |  |  |  |  |  |  |  |  |  |  |  |
| **PH** | -0.236  ** | 1.000 |  |  |  |  |  |  |  |  |  |  |  |  |  |  |
| **TN** | -0.162  * | 0.241  ** | 1.000 |  |  |  |  |  |  |  |  |  |  |  |  |  |
| **PN** | -0.010 | 0.275  *** | 0.818  *** | 1.000 |  |  |  |  |  |  |  |  |  |  |  |  |
| **FLL** | -0.332  *** | 0.367  *** | 0.344  *** | 0.259  ** | 1.000 |  |  |  |  |  |  |  |  |  |  |  |
| **FLW** | -0.206  * | 0.545  *** | 0.135  * | 0.165  * | 0.338  *** | 1.000 |  |  |  |  |  |  |  |  |  |  |
| **PL** | -0.095 | 0.586  *** | 0.100 | 0.120 | 0.478  *** | 0.427  *** | 1.000 |  |  |  |  |  |  |  |  |  |
| **BM** | -0.055 | 0.670  *** | 0.585  *** | 0.612  *** | 0.344  *** | 0.361  *** | 0.322  *** | 1.000 |  |  |  |  |  |  |  |  |
| **SER** | -0.564  *** | 0.623  *** | 0.256  *** | 0.150 | 0.463  *** | 0.312  *** | 0.334  *** | 0.461  *** | 1.000 |  |  |  |  |  |  |  |
| **HI** | -0.206  * | 0.180  * | -0.129 | -0.052 | 0.121 | 0.238  ** | 0.147 | -0.227  ** | 0.196  * | 1.000 |  |  |  |  |  |  |
| **GW** | -0.154 | 0.594  *** | 0.076 | 0.123 | 0.227  ** | 0.488  *** | 0.512  *** | 0.312  *** | 0.190  * | 0.154 | 1.000 |  |  |  |  |  |
| **LSL_1_** | -0.406  *** | 0.633  *** | 0.207  * | 0.120 | 0.235  ** | 0.355  *** | 0.391  *** | 0.419  *** | 0.581  *** | 0.189  * | 0.346  *** | 1.000 |  |  |  |  |
| **LSL_2_** | -0.410  *** | 0.722  *** | 0.199  * | 0.187  * | 0.294  *** | 0.374  *** | 0.404  *** | 0.454  *** | 0.627  *** | 0.135 | 0.314  *** | 0.533  *** | 1.000 |  |  |  |
| **LSL_3_** | -0.424  *** | 0.552  *** | 0.143 | 0.155 | 0.256  ** | 0.402  *** | 0.284  *** | 0.368  *** | 0.500  *** | 0.274  *** | 0.317  *** | 0.426*** | 0.505  *** | 1.000 |  |  |
| **GY** | -0.124 | 0.576  *** | 0.254  ** | 0.357  *** | 0.289  *** | 0.395  *** | 0.306  *** | 0.426  *** | 0.445  *** | 0.701  *** | 0.288  *** | 0.395  *** | 0.365  *** | 0.426  *** | 1.000 |  |
| **SR** | -0.244  ** | 0.609  *** | 0.254  ** | 0.383  *** | 0.299  *** | 0.405  *** | 0.416  *** | 0.475  *** | 0.390  *** | 0.136 | 0.466  *** | 0.400  *** | 0.426  *** | 0.391  *** | 0.425  *** | 1.000 |

| * Significant at P ≤ 0.05 |
| --- |
| ** Significant at P ≤ 0.01 |
| *** Significant at P ≤ 0.001 |
