# Supplementary material for: Identification of QTLs for yield and agronomic traits in rice under stagnant flooding conditions
Source: Rice (N Y). 2017 Apr 20;10:15. doi: 10.1186/s12284-017-0154-5 (PMC5398972; doi:10.1186/s12284-017-0154-5)
Supplement: Supplementary file 7 — Correlations among traits under irrigated control condition. (DOCX 15 kb) [file 12284_2017_154_MOESM7_ESM.docx]

Supplementary Table 4b: Correlations among traits under irrigated control condition.

| **Trait** | **DTF** | **PH** | **TN** | **PN** | **FLL** | **FLW** | **PL** | **BM** | **SER** | **HI** | **GW** | **LSL_1_** | **LSL_2_** | **LSL_3_** | **GY** |
| --- | --- | --- | --- | --- | --- | --- | --- | --- | --- | --- | --- | --- | --- | --- | --- |
| **DTF** | 1.000 |  |  |  |  |  |  |  |  |  |  |  |  |  |  |
| **PH** | 0.079 | 1.000 |  |  |  |  |  |  |  |  |  |  |  |  |  |
| **TN** | 0.095 | -0.324  *** | 1.000 |  |  |  |  |  |  |  |  |  |  |  |  |
| **PN** | 0.070 | -0.265  *** | 0.815  *** | 1.000 |  |  |  |  |  |  |  |  |  |  |  |
| **FLL** | 0.091 | 0.208  * | -0.072 | -0.018 | 1.000 |  |  |  |  |  |  |  |  |  |  |
| **FLW** | -0.449  *** | 0.188  * | -0.169  * | -0.134 | 0.130 | 1.000 |  |  |  |  |  |  |  |  |  |
| **PL** | -0.255  ** | 0.359  *** | -0.189  * | -0.066 | 0.476  *** | 0.205  * | 1.000 |  |  |  |  |  |  |  |  |
| **BM** | 0.493  *** | 0.186  * | 0.440  *** | 0.401  *** | 0.137 | -0.1487 | -0.012 | 1.000 |  |  |  |  |  |  |  |
| **SER** | -0.507  *** | 0.347  *** | -0.092 | -0.034 | 0.241  ** | 0.398  *** | 0.423  *** | -0.227  ** | 1.000 |  |  |  |  |  |  |
| **HI** | -0.488  *** | -0.087 | -0.326  *** | -0.312  *** | -0.069 | 0.138 | 0.100 | -0.712  *** | 0.284  *** | 1.000 |  |  |  |  |  |
| **GW** | -0.133 | 0.295  *** | -0.375  *** | -0.333  *** | -0.001 | 0.161  * | 0.187  * | -0.083 | 0.104 | 0.078 | 1.000 |  |  |  |  |
| **LSL_1_** | 0.348  *** | 0.618  *** | -0.157  * | -0.166  * | 0.092 | -0.117 | -0.028 | 0.259  ** | 0.042 | -0.151 | 0.152 | 1.000 |  |  |  |
| **LSL_2_** | -0.334  *** | 0.494  *** | -0.191  * | -0.198  * | 0.067 | 0.200  * | 0.315  *** | -0.064 | 0.431  *** | 0.229  ** | 0.156 | 0.107 | 1.000 |  |  |
| **LSL_3_** | -0.106 | 0.589  *** | -0.260  ** | -0.150 | 0.013 | 0.254  ** | 0.170  * | -0.066 | 0.268  *** | 0.1 | 0.286  *** | 0.201  * | 0.199  * | 1.000 |  |
| **GY** | -0.245  ** | 0.063 | -0.066 | -0.079 | 0.028 | 0.029 | 0.103 | -0.056 | 0.174  * | 0.707  *** | 0.033 | 0.036 | 0.253  ** | 0.073 | 1.000 |

| * Significant at P ≤ 0.05 |
| --- |
| ** Significant at P ≤ 0.01 |
| *** Significant at P ≤ 0.001 |
